# Transglycosylated Starch Modulates the Gut Microbiome and Expression of Genes Related to Lipid Synthesis in Liver and Adipose Tissue of Pigs

**DOI:** 10.3389/fmicb.2018.00224

**Published:** 2018-02-13

**Authors:** Monica A. Newman, Renée M. Petri, Dietmar Grüll, Qendrim Zebeli, Barbara U. Metzler-Zebeli

**Affiliations:** ^1^Institute of Animal Nutrition and Functional Plant Compounds, Department for Farm Animals and Veterinary Public Health, University of Veterinary Medicine Vienna, Vienna, Austria; ^2^Agrana Research & Innovation Center GmbH, Tulln, Austria

**Keywords:** pig, resistant starch type 4, gut microbiota, microbial metabolites, lipid metabolism, incretins, satiety, gene expression

## Abstract

Dietary inclusion of resistant starches can promote host health through modulation of the gastrointestinal microbiota, short-chain fatty acid (SCFA) profiles, and lipid metabolism. This study investigated the impact of a transglycosylated cornstarch (TGS) on gastric, ileal, cecal, proximal-colonic, and mid-colonic bacterial community profiles and fermentation metabolites using a growing pig model. It additionally evaluated the effect of TGS on the expression of host genes related to glucose and SCFA absorption, incretins, and satiety in the gut as well as host genes related to lipid metabolism in hepatic and adipose tissue. Sixteen growing pigs (4 months of age) were fed either a TGS or control (CON) diet for 11 days. Bacterial profiles were determined via Illumina MiSeq sequencing of the V3–5 region of the 16S rRNA gene, whereas SCFA and gene expression were measured using gas chromatography and reverse transcription-quantitative PCR. *Megasphaera*, which was increased at all gut sites, began to benefit from TGS feeding in gastric digesta, likely through cross-feeding with other microbes, such as *Lactobacillus*. Shifts in the bacterial profiles from dietary TGS consumption in the cecum, proximal colon, and mid colon were similar. Relative abundances of *Ruminococcus* and unclassified *Ruminococcaceae* genus were lower, whereas that of unclassified *Veillonellaceae* genus was higher in TGS- compared to CON-fed pigs (*p* < 0.05). TGS consumption also increased (*p* < 0.05) concentrations of SCFA, especially propionate, and lactate in the distal hindgut compared to the CON diet which might have up-regulated *GLP1* expression in the cecum (*p* < 0.05) and mid colon compared to the control diet (*p* < 0.10). TGS-fed pigs showed increased hepatic and decreased adipocyte expression of genes for lipid synthesis (*FASN, SREBP1*, and *ACACA*) compared to CON-fed pigs, which may be related to postprandial portal nutrient flow and reduced systemic insulin signaling. Overall, our data show that TGS consumption may affect gastrointestinal bacterial signaling, caused by changes in gut bacterial profiles and the action of propionate, and host lipid metabolism.

## Introduction

The gut microbiota has a significant impact on host metabolism, and may contribute to a variety of metabolic disorders such as obesity, insulin resistance, and cardiovascular disease (Boulangé et al., 2016). Extensive research has shown that diet can modulate the composition and function of the gut microbial community (Flint et al., 2015). Among the nutritional-based strategies used to modulate the gastrointestinal microbiota, the use of dietary fiber, including resistant starches (RS), is well-known (Conlon and Bird, 2015; Holscher, 2017). RS includes starch and starch degradation products that escape endogenous digestion in the small intestine and pass to the large intestine to be fermented by microbes (Englyst and Cummings, 1985). Dietary inclusion of various RS have been shown to alter the microbial community and fermentation intensity in the cecal and colonic digesta of pigs and in human feces, however, results diverged based upon gut site and the specific RS (Martínez et al., 2010; Walker et al., 2011; Haenen et al., 2013; Metzler-Zebeli et al., 2015b; Sun et al., 2016), indicating the importance of evaluating each RS and its effects at each gut site individually.

There are currently five classifications of RS based upon the properties they possess that cause resistance to endogenous enzymatic digestion in the small intestine (Birt et al., 2013).

The first three types (RS1-3) have been investigated to a much greater extent than the remaining types (RS4-5). Type 4 RS (RS4) encompasses RS that are chemically modified via transglycosylation, esterification, or crosslinking (Singh et al., 2007, 2010). Due to its differing molecular structure, different bacteria are capable of utilizing RS4, which causes divergent changes in the gut bacterial community compared to RS2 and RS3. For example, RS2 and RS3 have been shown to increase many butyrate-producing bacteria in the hindgut and feces of pigs such as *Ruminococcus, Lachnospiraceae, Faecalibacterium, Blautia*, and *Coprococcus* (Haenen et al., 2013; Sun et al., 2015; Umu et al., 2015), whereas RS4 increased other starch-degrading bacteria such as *Oscillibacter* and *Meniscus* (Metzler-Zebeli et al., 2015b). Likewise, in a direct comparison, RS2 and RS4 elicited different modulatory abilities on the human fecal bacterial microbiota (Martínez et al., 2010).

Increased fermentation and short-chain fatty acid (SCFA) production provides an important link between dietary RS consumption and host metabolism (den Besten et al., 2013; Haenen et al., 2013; Upadhyaya et al., 2016). The various RS differentially enhance acetate, propionate, butyrate, and valerate in the cecum, colon, and feces of pigs (Haenen et al., 2013; Newman et al., 2016). SCFA have profound effects on gut health as energy sources, inflammation modulators, vasodilators, and on gut motility (Tremaroli and Bäckhed, 2012). Evidence is also emerging that SCFA play a regulatory role in local, intermediary, and peripheral metabolism (Morrison and Preston, 2016). After being absorbed, propionate is primarily taken up by the liver and used as substrate for gluconeogenesis, whereas acetate is mostly utilized for lipogenesis in adipocytes (Morrison and Preston, 2016). Accordingly, dietary RS4 inclusion has been shown to affect lipid metabolism in pigs, humans, and rodents (Shimotoyodome et al., 2010, 2011; Metzler-Zebeli et al., 2015a; Newman et al., 2017). SCFA influence gene expression through inhibition of histone deacetylase and by binding to fatty acid-sensing G-protein-coupled receptors (McKenzie et al., 2017). These receptors play crucial roles in the promotion of gut homeostasis and modulate intestinal secretion of peptide YY (PYY), gastric inhibitory polypeptide (GIP), and glucagon-like peptide 1 (GLP-1; Zhou et al., 2008; Miyauchi et al., 2010; Nøhr et al., 2013), which have been linked to increased satiety in pigs and humans.

Available data regarding the effects of RS4 focus on the large intestine, leaving a considerable dearth of knowledge regarding its effects on the microbial community and host interactions in the stomach and small intestine. We recently showed that dietary inclusion of a transglycosylated starch (TGS) product altered the acetate and propionate profiles in the blood (Newman et al., 2017). However, its effects on the gut bacterial community profiles and SCFA signaling and transport have not been evaluated thus far. Therefore, this study investigated the impact of TGS on gastric, ileal, cecal, proximal-colonic, and mid-colonic bacterial community profiles and fermentation metabolites in growing pigs. We additionally evaluated its effect on the expression of host genes related to glucose and SCFA absorption, incretins, and satiety in the gut as well as host genes related to lipid metabolism in hepatic and adipose tissue. We hypothesized that dietary inclusion of TGS would alter bacterial community profiles throughout the gastrointestinal tract, thereby altering hindgut fermentation metabolite profiles. Additionally, due to the expected changes in blood insulin, SCFA, and lipids that were seen in a previous study regarding the effects of this TGS product (Newman et al., 2017), we hypothesized decreased expression of incretin genes and increased expression of genes related to glucose and SCFA transport, satiety, and lipid oxidation with TGS consumption. Pigs were used as a model for humans in this study because they are seen as a reliable model to study digestive physiology, metabolic responses, and dietary modulation of the gut microbiota (Guilloteau et al., 2010; Heinritz et al., 2013).

## Materials and Methods

### Ethics Statement

All procedures that involved animal handling were approved by the institutional ethics committee of the University of Veterinary Medicine Vienna (Vienna, Austria) and the national authority according to to paragraph 8 of the Law for Animal Experiments, Tierversuchsgesetz – TVG (GZ 68.205/0051-II/3b/2013).

### Animals, Housing, and Experimental Design

Sixteen crossbred growing pigs [(Landrace × Large White) × Piétrain; BW = 45.4 ± 4.24 kg; age = 4 months] were used in this study. Four days prior to the start of the experiment, pigs were moved into individual metabolism pens (1.0 m × 1.2 m) for environmental adaptation, where they were housed for the duration of the experiment. Pens were made of Plexiglas walls and completely slatted flooring and were cleaned daily. Each pen was equipped with a single-space feeder and a nipple drinker for *ad libitum* access to demineralized water. Pigs were housed in an environmentally controlled room, and room temperature was checked twice daily to ensure optimal temperature for the pigs. After the environmental adaptation period, pigs were randomly allotted to 1 of 2 dietary treatments in a completely randomized design with two 11-day replicate batches. Each replicate batch consisted of an 8-day dietary acclimation period, followed by 2 days of fecal collection to determine the apparent total tract digestibility of nutrients, and 1 day of serial slaughter. Four pigs were allotted per diet in each of the two replicate batches, which provided a total of eight observations per dietary treatment.

### Diets

During the environmental adaptation period, pigs consumed a commercial grower diet (metabolizable energy = 3.19 Mcal/kg; crude protein = 16.8%, as-fed basis). After this period, pigs were switched to 1 of the 2 experimental diets, which consisted of 72.1% purified cornstarch, 18.0% casein, 4.0% lignocellulose (FibreCell M1; agromed Austria GmbH, Kremsmünster, Austria), 1.0% rapeseed oil, 4.0% monocalcium phosphate, and 0.6% vitamin-mineral premix (Newman et al., 2017; Supplementary Table S1). Diets were formulated to meet or exceed current nutrient requirements for growing pigs (National Research Council, 2012). To evaluate the effect of TGS on apparent total tract digestibility of nutrients titanium dioxide (0.3%) was included as an indigestible marker. The 2 experimental diets were identical in their ingredient composition, except for the starch component. The starch used in the control diet (CON) was a rapidly digestible waxy cornstarch (Agrana Research and Innovation Center GmbH (ARIC), Tulln, Austria), whereas in the test diet (TGS) 50% of the native waxy cornstarch was replaced by a transglycosylated waxy cornstarch (ARIC). The TGS product was prepared via an acid-catalyzed transglycosylation of the native waxy cornstarch, which rearranges the glycosidic bonds that are present. Native waxy cornstarch has two types of glycosidic bonds, α(1,4) and α(1,6). The TGS product, due to the acid-catalyzed transglycosylation of the waxy cornstarch, has eight types of glycosidic bonds: α(1,2), α(1,3), α(1,4), α(1,6), β(1,2), β(1,3), β(1,4), and β(1,6). The analyzed nutrient composition of the diets is presented in Supplementary Table S1. Feed was offered *ad libitum* 3 times daily at 8:00, 12:00, and 16:30 h. At feeding, the experimental diets were mixed with water in a ratio of about 2:1 and immediately offered to the pigs. Feed allowances were calculated to exceed the pigs’ appetites. Feed leftovers (feed spillage and feed remaining in the feeding bowls) were collected, dried, and weighed to determine dry matter intake.

### Sample Collection

Fresh fecal samples were collected from the slatted flooring and trays beneath the cages on days 9 and 10 via grab sampling to determine apparent total tract digestibility coefficients of dietary nutrients. Subsamples of freshly defecated feces were immediately frozen at -20°C until analysis. Approximately 3 h after feeding on day 11 of each replicate batch, pigs were anesthetized via an intramuscular injection of 10 ml/kg body weight ketamine HCl (Narketan; Vétoquinol AG, Ittigen, Austria) and 3 ml/kg body weight azaperone (Stresnil; Biokema SA, Crissier, Switzerland). Blood samples were collected via cardiac puncture into serum collection tubes (S-Monovette 9.0 mL Z; Sarstedt AG & Co., Nümbrecht, Germany), placed on ice until they were centrifuged at 1,811 × *g* for 10 min (Eppendorf Centrifuge 5810 R, Eppendorf, Hamburg, Germany), and frozen at -20°C for later analysis. Pigs were then immediately euthanized via intracardiac injection of embutramide (T61; 10 mL/kg body weight; MSD Animal Health, Vienna, Austria). After euthanasia the gastrointestinal tract, liver, and a subcutaneous abdominal fat sample from the ventral abdominal wall (level with the last rib) were removed. A 5-cm^2^ tissue piece from the caudate lobe of the liver and an abdominal fat sample were immediately washed in phosphate-buffered saline, blotted dry with paper towel, cut into small pieces, snap frozen in liquid nitrogen, and stored at -80°C for RNA isolation. Meanwhile, the intestines were dissected from the mesentery and each gut site (stomach, duodenum, jejunum, ileum, cecum, proximal colon, and mid colon) was tied off to prevent the mixing of digesta between gut sites. In order to identify the mid-colonic region, the colon was divided into three equal parts. To identify the mid jejunum, the small intestine was divided in half. Digesta from the stomach, ileum (the last 30 cm of the small intestine), cecum, proximal colon, and mid colon were collected aseptically after being opened at the mesentery. Digesta from each gut site was homogenized and subsampled. Digesta subsamples for microbiota analysis were immediately snap frozen in liquid nitrogen and stored at -80°C until analysis. Digesta subsamples for SCFA and lactate were placed on ice until they could be frozen at -20°C. Tissue samples from the duodenum, mid jejunum, ileum, cecum, proximal, and mid colon were washed in ice-cold phosphate-buffer. Mucosal scrapings were then collected from 20-cm tissue sections with a glass microscope slide, immediately snap frozen in liquid nitrogen, and stored at -80°C until analysis.

### DNA Isolation, Library Preparation, and Illumina MiSeq Sequencing

Total DNA was extracted from 250 mg of gastric, ileal, cecal, proximal-colonic, and mid-colonic digesta samples using a PowerSoil DNA isolation kit (Mo Bio Laboratories, Carlsbad, CA, United States) according to the manufacturer’s instructions with slight modifications (Metzler-Zebeli et al., 2015b). To ensure proper lysis of bacteria, samples were heated at 70°C for 10 min as an additional step between mixing the digesta samples with C1 buffer and bead beating. The DNA concentration was measured with a Qubit 2.0 fluorometer (Life Technologies, Carlsbad, CA, United States) using the Qubit double-stranded DNA HS assay kit (Life Technologies, Carlsbad, CA, United States). DNA extracts were sent to Microsynth (Balgach, Switzerland) for 16S rRNA gene PCRs, library preparation, and sequencing using the Illumina MiSeq sequencing platform (Illumina Inc., San Diego, CA, United States). The V3–5 hypervariable regions of bacterial 16S rRNA genes were amplified using the primers 357F-HMP (5′-CCTACGGGAGGCAGCAG-3′) and 926R-HMP (5′-CCGTCAATTCMTTTRAGT-3′) to produce an amplicon size of approximately 523 bp (Peterson et al., 2009). Libraries were constructed by ligating sequencing adapters and indices onto purified PCR products using the Nextera XT sample preparation kit (Illumina Inc.). Equimolar quantities of each library was pooled and sequenced on an Illumina MiSeq sequencing platform using a 300 bp read length paired-end protocol. After sequencing, the overlapping paired-end reads were stitched, trimmed, and quality-filtered by Microsynth.

### Sequence Processing and Analysis

A total of 6,016,953 sequences with a mean Phred score of 29 to 35 were attained from Microsynth and were subsequently processed using QIIME (Caporaso et al., 2010). Reads were merged using the demultiplexing method, low quality sequences were removed (*q* < 20), and chimeric sequences were filtered out using USEARCH81 and the gold.fa database (Edgar, 2010). Reads were then aligned using the Greengenes database (version 13.8) and sequences were clustered into operational taxonomic units (OTUs) using a 16S rRNA distance of 0.03. All OTUs with less than 10 sequences were removed, which resulted in 3,890 OTUs for downstream analysis. Microbial richness and diversity were calculated using the non-parametric species estimator Chao1 and the Shannon and Simpson diversity indices. To determine the degree of similarity between samples, weighted and unweighted Unifrac distance matrices were calculated and used to generate principal co-ordinates analyses (PCoA) plots. The raw sequence reads were uploaded to the NCBI BioProject databank under the BioProject number: PRJNA396828.

### RNA Isolation and Quantitative Real-Time PCR

Total RNA was isolated from jejunal, ileal, cecal, and mid-colonic mucosal scrapings as well as hepatic tissue and abdominal fat. Tissue samples (20 mg) were combined with lysis buffer (RNeasy Mini QIAcube kit, Qiagen, Hilden, Germany) and autoclaved ceramic beads (0.6 g; 1.4 mm; VWR). Samples were homogenized using the FastPrep-24 instrument (MP Biomedicals, Santa Ana, CA, United States). The remainder of the RNA isolation protocol was completed according to manufacturer’s instructions using the automated QIAcube robotic workstation (Qiagen, Hilden, Germany). After extraction, all samples were treated with the Turbo DNA kit (Life Technologies Limited, Vienna, Austria) to remove genomic DNA. The RNA was quantified using a Qubit HS RNA Assay kit on the Qubit 2.0 Fluorometer (Life Technologies), and the quality of isolated RNA was evaluated with the Agilent Bioanalyzer 2100 (Agilent RNA 6000 Nano Assay, Agilent Technologies, Waghaeusel-Wiesental, Germany). The RNA integrity numbers (RIN) ranged from 7.3 to 9.9 in most samples. Only two jejunal samples had a RIN of 6.1 and 6.4. Complementary DNA was synthesized from 2 μg of RNA using the High Capacity cDNA RT kit (Life Technologies Limited, Vienna, Austria) and 1 μl of RNase inhibitor (Biozym, Hessisch Oldendorf, Germany) was added to each reaction.

Primers utilized for quantitative PCR (qPCR) are listed in Supplementary Table S2. The primers were designed using and, together with previously published primers, were verified with Primer-BLAST^1^ and tested for efficiencies and specificity using melting curve analysis (Supplementary Table S2). Amplifications were performed on a real-time PCR Mx3000P thermocycler (Agilent Technologies) using the following conditions: 95°C for 5 min, followed by 95°C for 10 s, 60°C for 30 s, and 72°C for 30 s for 40 cycles, followed by the generation of melting curves. Negative controls and reverse transcription controls (RT minus) were included in order to control for residual DNA contamination. Each 20 μl reaction consisted of 50 ng cDNA, 10 μl Fast Plus Eva Green master mix with low ROX (Biotium, Hayward, CA, United States), 200 nM each of forward and reverse primers, and DEPC-treated water in a 96 well plate (VWR, Vienna, Austria). All reactions were run in duplicate.

Five housekeeping genes (HKG) were analyzed and the three best-fit were selected using NormFinder (Andersen et al., 2004) and BestKeeper (Pfaffl et al., 2004). The geometric mean expression level of the three most stably expressed HKG (*ACTB, B2M, GAPDH*) was used for normalization of target gene expression levels. For this, the mean raw gene expression data, obtained as *C*_q_ values, of the identified HKG were subtracted from the *C*_q_ of the target genes to determine Δ*C*_q_ values. Relative gene expression was calculated relative to the pig with the lowest expression of the respective gene using the 2^-ΔΔ*C*q^ method (Livak and Schmittgen, 2001).

### Chemical Analyses

All feed and fecal samples were analyzed in duplicate for dry matter, gross energy, protein, total starch, calcium, phosphorus, ash, and titanium dioxide as recently described (Newman et al., 2017). Apparent total tract digestibility coefficients were calculated for all dietary nutrients according to Oresanya et al. (2007). Total lactate concentrations in fecal samples were determined using a commercially available kit (K-DLATE, Megazyme International, Wicklow, Ireland). The pH of gastric, ileal, cecal, proximal-colonic, and mid-colonic digesta was measured using a Beckman Φ63 pH meter (Beckman Coulter, Fullerton, CA, United States). Individual SCFA concentrations in feces were determined using gas chromatography as recently described (Metzler-Zebeli et al., 2015b).

### Biochemical Variables, Acute Phase Proteins, and Brush Border Enzymes

Serum glucose, urea, cholesterol, triglycerides, and non-esterified fatty acids were measured via standard enzymatic colorimetric analysis using an autoanalyzer for clinical chemistry (Cobas 6000/c501; Roche Diagnostics GmbH, Vienna, Austria). A porcine-specific commercial ELISA kit was used to analyze serum concentration of haptoglobin (Genway, San Diego, CA, United States) according to the manufacturer’s instructions. The activity of lactase, maltase, and sucrase was determined in mucosal scrapings from the duodenum. Preparation of duodenal homogenates (20%, w/v) and mucosal enzyme activity measurements were performed as recently described (Metzler-Zebeli et al., 2017).

### Statistical Analyses

This study was designed as a completely randomized design with two dietary treatments in two replicate batches with four pigs per treatment in each batch. The UNIVARIATE procedure in SAS (Version 9.4, SAS Institute Inc., Cary, NC, United States) was used to verify normality and homogeneity of variances. The data were normally distributed. To compare differences between diets, data were subjected to ANOVA using the MIXED procedure in SAS. Data were analyzed using the fixed effect of diet and the random effect of replicate batch in the main model with individual pig considered as the experimental unit. Degrees of freedom were approximated using the Kenward–Rogers method (ddfm = kr). The pairwise comparisons among least-square means were performed using the Tukey-Kramer test. All *p-*values from ANOVA and multiple comparison analyses of the most abundant bacterial genera were adjusted by the false discovery rate (FDR) (Benjamini and Hochberg, 1995) using the MULTTEST procedure (SAS). FDR-corrected *p-*values below 0.05 were considered significant and as tendencies if 0.05 ≤*p* < 0.10. Pearson’s correlation analysis using the PROC CORR procedure of SAS was used to establish and quantify the relationships among microbial metabolites, gene expression, and bacterial genera data. Correlations were considered significant if *p* < 0.05. For visualization, correlation matrices were then generated using the packages ‘corrplot’ (Wei, 2010) and ‘ggplot2’ (Wickham, 2009) in R Studio (version 1.0.136).

## Results

### Pig Performance, Nutrient Digestibility, and Serum Parameters

All pigs were clinically healthy during the experiment as intestinal and systemic disorders were absent. No differences were observed in feed intake or average daily gain between dietary treatments, but TGS-fed pigs tended to have a lower feed to gain ratio compared to CON-fed pigs (*p* < 0.10, Supplementary Table S3). The TGS diet reduced apparent total tract digestibility of dry matter, gross energy, starch, and crude protein compared to the CON diet (*p* < 0.05, Supplementary Table S3), but had no effect on the apparent total tract digestibility of calcium, phosphorus, or crude ash. Brush border enzyme activities at the duodenal mucosa showed a trend for lower lactase activity in TGS- compared to CON-fed pigs (*p* < 0.10, Supplementary Table S4). Blood serum parameters were not significantly affected by dietary treatment (*p* > 0.10, Supplementary Table S4).

### General Bacterial Structure and Community Composition

**FIGURE 1:**
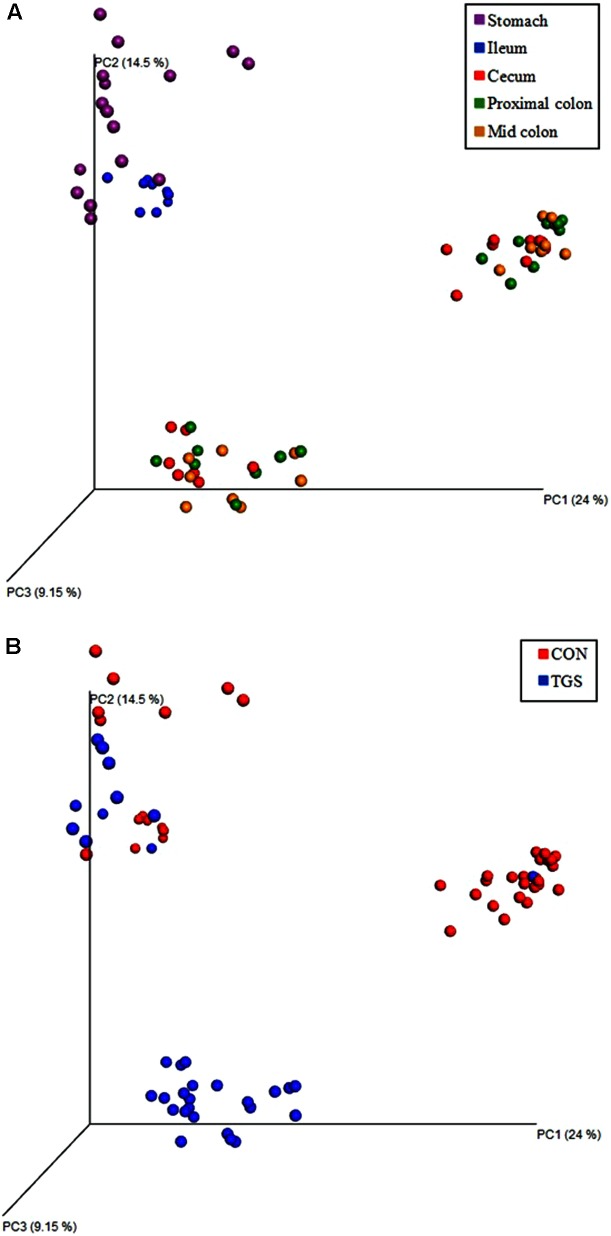
Principal co-ordinates analysis of unweighted Unifrac distances for digesta-associated microbiota **(A)** by gut site and **(B)** by dietary treatment of pigs fed the control (CON) or transglycosylated starch (TGS) diet.

**FIGURE 2:**
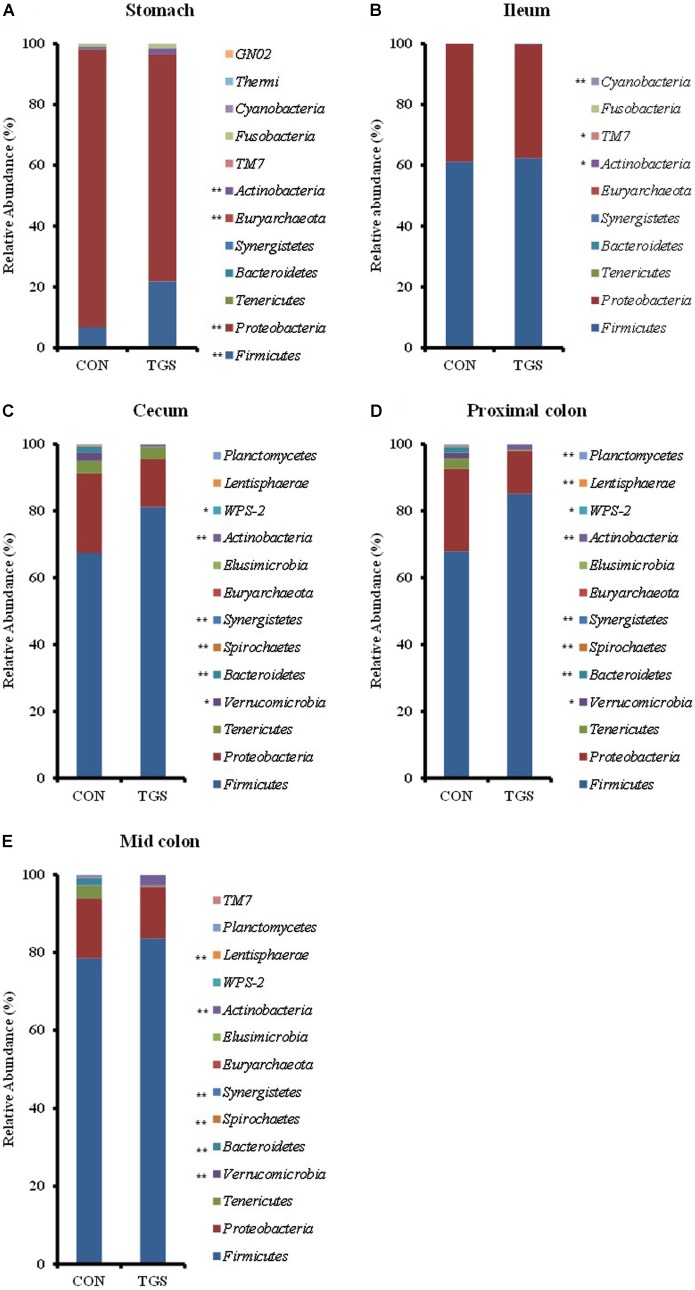
Microbiome composition at the phylum level for 16S rRNA sequences in gastric **(A)**, ileal **(B)**, cecal **(C)**, proximal-colonic **(D)**, and mid-colonic **(E)** digesta of pigs fed transglycosylated (TGS) or control (CON) starch diets. Values are presented as least square means ± SEM; *n* = 8 pigs per dietary treatment for stomach, cecum, proximal colon, and mid colon; *n* = 7 pigs in the CON diet group and *n* = 4 pigs in the TGS diet group for ileum. ^∗∗^CON and TGS differ within the intestinal segment, *P* < 0.05; ^∗^CON and TGS tend to differ within the intestinal segment, 0.05 ≤*P* ≤ 0.10.

Unweighted Unifrac analysis indicated that gastric and ileal samples clustered separately from those of the large intestine (**Figure 1A**). The two most dominant phyla at all five gut sites were *Firmicutes* and *Proteobacteria*, which together accounted for 93 to 99% of all sequences (**Figure 2**). Across all samples, *Proteobacteria* (83.0%) were the most dominant phyla in the stomach followed by *Firmicutes* (14.3%). However, *Firmicutes* predominated the ileum (61.8%), cecum (74.3%), proximal colon (76.5%), and mid colon (81.0%), which occurred mainly at the expense of *Proteobacteria* (38.1% ileum, 19.1% cecum, 18.9% proximal colon, 14.4% mid colon). In the hindgut, *Tenericutes* also played a significant role in the general community composition, comprising 3.6, 1.6, and 1.7% of phyla present in the cecum, proximal colon, and mid colon, respectively. All other phyla were present at less than 1.5% relative abundance over all samples at each gut site. At genus level, unclassified *Enterobacteriaceae* (36.2%) and *Actinobacter* (18.6%) were among the most abundant in the stomach (Supplementary Table S5). The most abundant OTU belonging to unclassified *Enterobacteriaceae* was blasted against the Greengenes database^2^ and linked with its closest reference strain, which was *Escherichia* sp. *str. II_B13* (100.0% similarity). In the ileum *Turicibacter* (48.8%), unclassified *Enterobacteriaceae* (31.0%), and unclassified *Clostridiaceae* (11.6%) were the most abundant (Supplementary Table S5). Through all three hindgut sites, unclassified *Veillonellaceae* (19.2–30.4%) and *Ruminococcus* (15.0–17.4%) were the most abundant across all samples (Supplementary Table S5). The most abundant OTU belonging to unclassified *Veillonellaceae* was also blasted against the Greengenes database to identify its closest reference strain, which was *Selenomonas* sp. *str. WG* (94.56% similarity).

### Diet-Related Differences in the Bacterial Community

**FIGURE 3:**
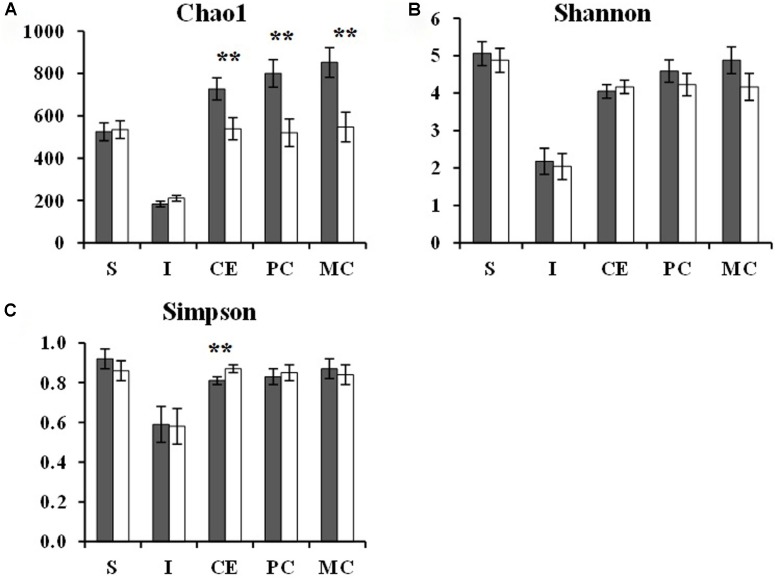
Chao1 richness estimate **(A)**, Shannon index **(B)**, and Simpson index **(C)** in gastric (S), ileal (I), cecal (CE), proximal-colonic (PC), and mid-colonic (MC) digesta of pigs fed the control (CON, 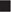) or transglycosylated starch (TGS, 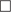) diet. Values are presented as least square means ± SEM; *n* = 8 pigs per dietary treatment for stomach, cecum, proximal colon, and mid colon; *n* = 7 pigs in the CON diet group and *n* = 4 pigs in the TGS diet group for ileum. ^∗∗^CON and TGS differ within the intestinal segment, *P* < 0.05.

Beta-diversity analysis showed separate clustering of cecal, proximal-colonic, and mid-colonic bacterial communities by diet (**Figure 1B**). Diet did not affect species richness in gastric or ileal digesta. However, TGS consumption led to 26, 35, and 36% lower (*p* < 0.05) Chao1 estimates in the cecal, proximal-colonic, and mid-colonic digesta, respectively, compared to the CON diet (**Figure 3**). Additionally, a 7% increase (*p* < 0.05) in the Simpson index was observed in the cecum of TGS- compared to CON-fed pigs (**Figure 3**). *Firmicutes* and *Proteobacteria*, the two most abundant phyla at every gut site, were only modified (*p* < 0.05) by TGS consumption in the stomach (**Figure 2**). The gastric digesta of TGS-fed pigs showed a 0.2-fold decrease in the relative abundance of *Proteobacteria* and a 2-fold increase in *Firmicutes* compared to CON-fed pigs. *Actinobacteria* was the only phylum whose relative abundance was affected at every gut site (2- to 10-fold increase, *p* < 0.10 at the ileum and *p* < 0.05 at all other gut sites) by TGS consumption. Relative abundances of *Synergistetes*, *Spirochaetes*, and *Bacteroidetes* decreased (*p* < 0.05) at all hindgut sites with the TGS diet compared to the CON diet. Additionally, the relative abundance of *Verrucomicrobia* tended (*p* < 0.10) to be lower in the cecum and proximal colon, and was lower (*p* < 0.05) in the mid colon of TGS- compared to CON-fed pigs. Other, less abundant phyla were also modified at the various gut sites by dietary TGS inclusion (**Figure 2**).

Of the 30 most abundant genera at each gut site, 4, 4, 6, 12, and 12 genera were affected (*p* < 0.05) and 4, 1, 6, 3, and 0 tended to be affected (*p* < 0.10) by TGS consumption in the stomach, ileum, cecum, proximal colon, and mid colon, respectively (**Tables 1**, **2**). Most genera affected by the TGS diet belonged to the phylum *Firmicutes*, except in the stomach where nearly half belonged to *Proteobacteria* and *Actinobacteria. Megasphaera* was the only genus that increased (4- to 11-fold, *p* < 0.10 in gastric digesta and *p* < 0.05 at all other gut sites) in relative abundance at all gut sites with TGS consumption. In gastric digesta, the most notable change was a 0.6-fold decrease (*p* < 0.05) in the second most abundant genus, *Actinobacillus*, with TGS consumption compared to the CON diet. Consumption of TGS also increased (*p* < 0.05) relative abundances of lesser abundant unclassified *Coriobacteriaceae* (20-fold), *Lactobacillus* (1-fold), and *Succiniclasticum* (13-fold) in gastric digesta. The ileum was the least affected gut site by dietary treatment, with less than 1% of ileal bacteria at both phylum and genus level altered by dietary TGS consumption. Changes in ileal digesta were to lesser abundant genera, including increased (*p* < 0.05) *Aggregatibacter*, unclassified *Veillonellaceae*, and *Mitsuokella* (6-, 4-, and 10-fold, respectively), as well as a tendency (*p* < 0.10) for increased unclassified *Clostridiales* (1-fold) with dietary TGS consumption. The cecum, proximal colon, and mid colon showed similar changes in bacterial profiles at both the phylum and genus level. Also, the beta diversity plots indicated clear clustering by diet (**Figure 1**). By far, the largest changes across the hindgut were to an unclassified *Veillonellaceae* genus (best blast hit on most abundant OTU: *Selenomonas*) and *Ruminococcus*. The relative abundance of *Ruminococcus* was decreased 0.9-fold in the cecum, proximal colon, and mid colon with TGS consumption, which was replaced by an increased relative abundance of unclassified *Veillonellaceae* in the cecum, proximal colon, and mid colon (41-, 33-, and 5-fold, respectively). Other notable changes that occurred across all hindgut sites included increased relative abundances of *Succiniclasticum* and an unclassified *Coriobacteriaceae* genus (best blast hit on most abundant OTU: *Olsenella*), and decreased relative abundances of an unclassified *Ruminococcaceae* genus (best blast hit on most abundant OTU: *Ruminococcus*), an unclassified *Bacteroidales* genus, and an unclassified *Desulfovibrionaceae* genus in TGS- compared to CON-fed pigs. The relative abundance of *Mitsuokella* was increased approximately 20-fold in the cecum (*p* < 0.10) and proximal colon (*p* < 0.05), respectively, whereas the relative abundance of *Akkermansia* tended (*p* < 0.10) to be decreased by approximately 1-fold at both gut sites in TGS- compared to CON-fed pigs. TGS consumption decreased (*p* < 0.05) the relative abundance of an unclassified *Clostridiales* genus and an unclassified *Clostridiaceae* genus 0.9-fold in both the proximal and mid colon. Additionally, the relative abundance of *Succinivibrio* tended to be 0.9-fold lower in the proximal colon of TGS- compared to CON-fed pigs. Other, less abundant genera were also modified at the various gut sites by dietary TGS inclusion (**Tables 1**, **2**).

**Table 1:** Differences in the 30 most abundant genera in gastric and ileal digesta of pigs fed transglycosylated (TGS) or control (CON) starch diets^1^.

	CON	TGS	SEM	*P*-value	FDR
**Stomach, %**					
*Actinobacillus*	26.09	11.02	3.630	0.011	0.033
*Megasphaera*	0.30	2.09	0.734	0.064	0.083
Unclassified *Coriobacteriaceae*	0.05	1.15	0.290	0.018	0.036
*Lactobacillus*	0.31	0.66	0.109	0.036	0.057
*Succiniclasticum*	0.04	0.55	0.126	0.014	0.033
Unclassified *Microbacteriaceae*	0.35	0.19	0.085	0.069	0.085
*Mannheimia*	0.33	0.12	0.112	0.070	0.085
Unclassified *Gemellaceae*	0.12	0.33	0.088	0.089	0.091
**Ileum, %**			
Unclassified *Clostridiales*	0.19	0.38	0.104	0.073	0.085
*Aggregatibacter*	0.03	0.20	0.071	0.025	0.045
Unclassified *Veillonellaceae*	0.03	0.16	0.047	0.039	0.058
*Megasphaera*	0.02	0.12	0.008	<0.0001	0.003
*Mitsuokella*	0.01	0.11	0.028	0.027	0.047


*^*1*^Data are presented as least square means ± SEM; n = 8 pigs per dietary treatment in the stomach; n = 7 pigs in the CON diet group and n = 4 pigs in the TGS diet group in the ileum. FDR, false discovery rate. Only values for the 30 most abundant genera per gut site that were different (P < 0.10) after FDR-correction are presented.*

**Table 2:** Differences in the 30 most abundant genera in hindgut digesta of pigs fed transglycosylated (TGS) or control (CON) starch diets^1^.

	CON	TGS	SEM	*P*-value	FDR
**Cecum, %**					
Unclassified *Veillonellaceae*	0.90	37.56	6.740	0.002	0.024
*Ruminococcus*	30.40	3.34	8.880	0.014	0.033
*Megasphaera*	0.63	7.52	1.799	0.017	0.035
Unclassified *Ruminococcaceae*	6.54	1.47	1.751	0.023	0.043
*Mitsuokella*	0.24	5.21	2.399	0.086	0.089
*Akkermansia*	2.52	0.12	0.881	0.075	0.085
*Succiniclasticum*	0.07	2.13	0.785	0.058	0.077
Unclassified *Bacteroidales*	1.70	0.25	0.612	0.040	0.058
Unclassified *Christensenellaceae*	0.90	0.32	0.219	0.079	0.087
*Oscillospira*	0.28	0.09	0.068	0.074	0.085
Unclassified *Desulfovibrionaceae*	0.26	0.09	0.097	0.084	0.089
Unclassified *Coriobacteriaceae*	0.02	0.31	0.084	0.029	0.049
**Proximal colon, %**					
Unclassified *Veillonellaceae*	1.54	52.09	6.728	<0.001	<0.001
*Ruminococcus*	27.46	2.56	6.022	0.005	0.024
Unclassified *Clostridiales*	11.35	1.02	4.036	0.014	0.033
Unclassified *Ruminococcaceae*	9.96	1.60	2.061	0.013	0.033
*Megasphaera*	1.06	6.58	1.195	0.006	0.024
*Succinivibrio*	3.54	0.24	1.336	0.085	0.089
*Mitsuokella*	0.17	3.45	1.241	0.012	0.033
*Succiniclasticum*	0.06	3.27	1.300	0.019	0.037
*Akkermansia*	1.71	0.02	0.608	0.069	0.085
Unclassified *Bacteroidales*	1.54	0.09	0.391	0.015	0.033
Unclassified *Coriobacteriaceae*	0.05	1.17	0.232	0.004	0.024
*Desulfovibrio*	0.83	0.38	0.101	0.007	0.024
Unclassified *Clostridiaceae*	1.10	0.06	0.283	0.013	0.033
*Phascolarctobacterium*	0.58	0.29	0.114	0.098	0.098
Unclassified *Desulfovibrionaceae*	0.40	0.08	0.108	0.005	0.024
**Mid colon, %**					
Unclassified *Veillonellaceae*	9.16	51.63	9.241	0.006	0.024
*Ruminococcus*	31.11	3.60	5.986	0.006	0.024
Unclassified *Ruminococcaceae*	14.61	2.58	2.386	0.003	0.024
Unclassified *Clostridiales*	12.74	1.02	4.693	0.031	0.050
*Megasphaera*	1.50	6.83	1.563	0.007	0.024
*Succiniclasticum*	0.13	3.63	1.225	0.038	0.058
Unclassified *Coriobacteriaceae*	0.06	2.00	0.605	0.003	0.024
Unclassified *Bacteroidales*	1.49	0.06	0.296	0.004	0.024
Unclassified *Clostridiaceae*	0.62	0.04	0.189	0.048	0.068
*Oscillospira*	0.33	0.05	0.069	0.015	0.033
Unclassified *Desulfovibrionaceae*	0.28	0.05	0.059	0.005	0.024
Unclassified *Alphaproteobacteria*	0.30	0.004	0.096	0.050	0.068


*^*1*^Data are presented as least square means ± SEM; n = 8 pigs per dietary treatment. FDR, false discovery rate. Only values for the 30 most abundant genera per gut site that were different (P < 0.10) after FDR-correction are presented.*

### Microbial Metabolites

**FIGURE 4:**
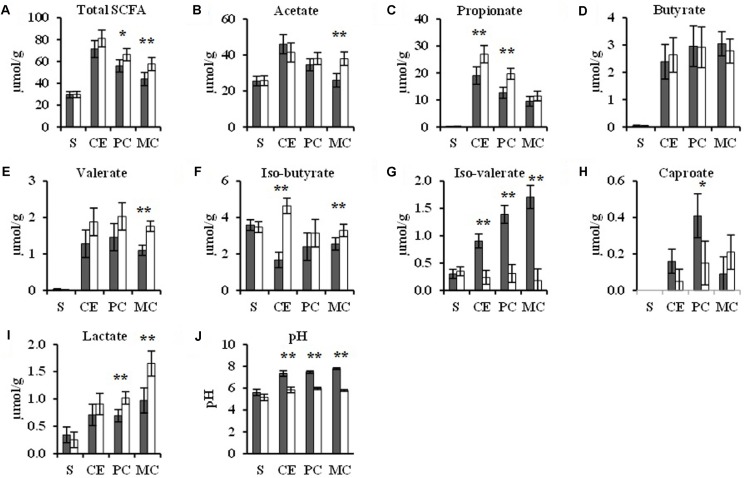
Total SCFAs **(A)**, acetate **(B)**, propionate **(C)**, butyrate **(D)**, valerate **(E)**, iso-butyrate **(F)**, iso-valerate **(G)**, caproate **(H)**, total lactate **(I)**, and pH values **(J)** in gastric (S), cecal (CE), proximal-colonic (PC), and mid-colonic (MC) digesta of pigs fed the control (CON, 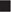) or transglycosylated starch (TGS, 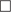) diet. Values are presented as least square means ± SEM; *n* = 8 pigs per dietary treatment. ^∗∗^CON and TGS differ within the intestinal segment, *P* < 0.05. ^∗^CON and TGS tend to differ within the intestinal segment, 0.05 < *P* < 0.10.

Total SCFA concentrations tended (*p* < 0.10) to be 0.2-fold greater in the proximal colon and were 0.3-fold higher (*p* < 0.05) in the mid colon with TGS consumption compared to the CON diet (**Figure 4**). Individual SCFA profiles showed 0.4-fold greater propionate and 1.8-fold greater iso-butyrate concentrations in the cecum as well as 0.6-fold more propionate in the proximal colon with TGS consumption compared to the CON diet (*p* < 0.05). In the mid colon, acetate, valerate, and iso-butyrate concentrations were increased by 0.5-, 0.6-, and 0.3-fold, respectively, in TGS- compared to CON-fed pigs (*p* < 0.05). Additionally, TGS-fed pigs showed an average of 0.8-fold lower (*p* < 0.05) iso-valerate concentrations in the cecum, proximal colon, and mid colon as well as a trend (*p* < 0.10) for a 0.6-fold lower caproate concentration in the proximal colon compared to the CON diet. No differences were observed in butyrate concentrations between dietary treatments. TGS-fed pigs also showed 0.5- and 0.7-fold greater lactate concentrations in the proximal colon and mid colon, respectively, compared to CON-fed pigs (*p* < 0.05). Furthermore, consumption of the TGS diet caused 0.2- to 0.3-fold lower (*p* < 0.05) pH in the cecum, proximal colon, and mid colon compared to the CON diet. Due to insufficient digesta at the terminal ileum in numerous pigs, data were not sufficient to provide SCFA or pH values at this gut site.

### Relative Expression of Target Genes in Gut Mucosa, Hepatocytes, and Adipocytes

**FIGURE 5:**
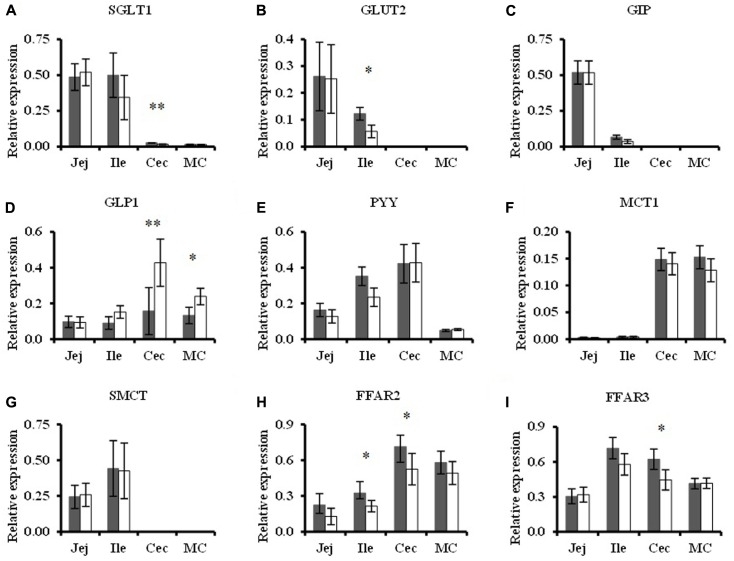
Relative expression of target genes in jejunal (Jej), ileal (Ile), cecal (Cec), and mid-colonic (C2) mucosa of pigs fed the control (CON, 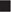) or transglycosylated starch (TGS, 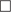) diet. Relative **(A)** *SGLT1*, **(B)** *GLUT2*, **(C)** *GIP*, **(D)** *GLP1*, **(E)** *PYY*, **(F)** *MCT1*, **(G)** *SMCT*, **(H)** *FFAR2*, and **(I)** *FFAR3* expression. Values are presented as least square means ± SEM; *n* = 8 per dietary treatment. ^∗∗^CON and TGS differ within the intestinal segment, *P* < 0.05. ^∗^CON and TGS tend to differ within the intestinal segment, 0.05 < *P* < 0.10.

Of the genes that were targeted at the intestinal mucosa, no differences were seen in the jejunum between dietary treatments. However, in the ileum the mucosal expression of *GLUT2* and *FFAR2* were down-regulated in TGS-fed pigs compared to CON-fed pigs by 0.5-fold (*p* < 0.10) and 0.3-fold (*p* < 0.05), respectively (**Figure 5**). The cecal mucosa of TGS-fed pigs showed a reduction (*p* < 0.05) in *SGLT1* (0.4-fold) expression, a tendency (*p* < 0.10) for reduced *FFAR2* (0.3-fold) and *FFAR3* (0.3-fold) expression, and increased (2-fold, *p* < 0.05) *GLP1* expression compared to CON-fed pigs (**Figure 5**). The TGS diet tended (*p* < 0.10) further to up-regulate mucosal expression of *GLP1* in the mid colon compared to the CON diet (0.8-fold, **Figure 5**). However, the mucosal expression of *PYY*, *GIP*, *MCT1*, and *SMCT* were similar between pigs fed TGS and CON diets at all gut sites.

**FIGURE 6:**
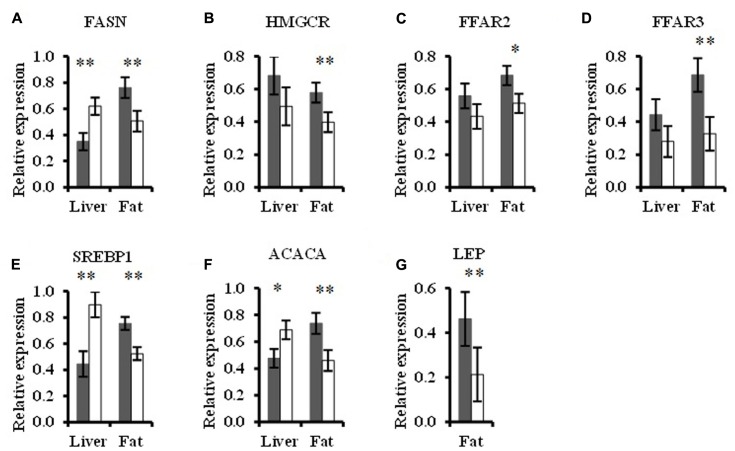
Relative expression of target genes in liver and abdominal fat samples that differed between pigs fed the control (CON, 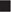) or transglycosylated starch (TGS, 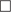) diet. Relative **(A)** *FASN*, **(B)** *HMGCR*, **(C)** *FFAR2*, **(D)** *FFAR3*, **(E)** *SREBP1*, **(F)** *ACACA*, and **(G)** *LEP* expression. Values are presented as least square means ± SEM; *n* = 8 per dietary treatment. ^∗∗^CON and TGS differ within the intestinal segment, *P* < 0.05. ^∗^CON and TGS tend to differ within the intestinal segment, 0.05 < *P* < 0.10.

In the liver, TGS consumption led to up-regulated (*p* < 0.05) *FASN* (0.8-fold), *SREBP1* (1-fold), and *ACACA* (0.5-fold, *p* < 0.10) expression compared to the CON diet (**Figure 6**). Relative expression of the remaining genes targeted in hepatocytes was similar between dietary treatments (**Figure 6** and Supplementary Figure S1).

Genes that were targeted in the abdominal fat showed down-regulated (*p* < 0.05) expression of *FASN* (0.3-fold), *HMGCR* (0.3-fold), *FFAR2* (0.3-fold, *p* < 0.10), *FFAR3* (0.5-fold), *SREBP1* (0.3-fold), *ACACA* (0.4-fold), and *LEP* (0.5-fold) with the TGS diet compared to the CON diet (**Figure 6**). Relative expressions of the remaining genes targeted in abdominal tissue were similar between dietary treatments (Supplementary Figure S1).

### Correlation Analysis

**FIGURE 7:**
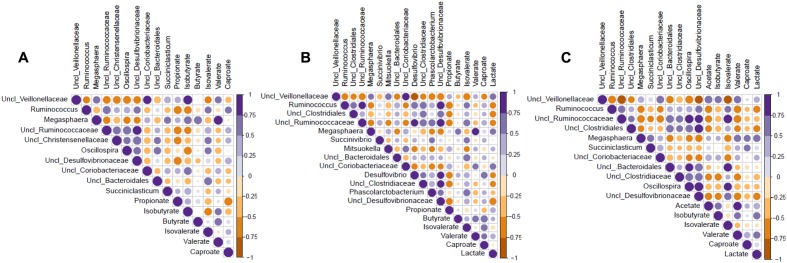
Correlation coefficients between microbial metabolites and bacterial genera in the cecum **(A)**, proximal colon **(B)**, and mid colon **(C)** of pigs fed control and transglycosylated starch diets. Uncl, unclassified.

Correlations between selected bacterial genera and microbial metabolites are presented in **Figures 7A–C**. Acetate was negatively correlated (*p* < 0.05) with 5 and positively correlated (*p* < 0.05) with 1 (unclassified *Veillonellaceae*) bacterial genera in the mid colon. In both the cecum and proximal colon, propionate was negatively correlated (*p* < 0.05) with *Ruminococcus*, unclassified *Ruminococcaceae*, and unclassified *Desulfovibrionaceae*. Propionate was also negatively correlated (*p* < 0.05) with 1 and 3 other genera in the cecum and proximal colon, respectively. Iso-butyrate was negatively correlated (*p* < 0.05) with 3 and 2 bacterial genera in the cecum and mid colon, respectively, as well as positively correlated (*p* < 0.05) with 4 (unclassified *Veillonellaceae*, *Megasphaera*, unclassified *Coriobacteriaceae*, and *Succiniclasticum*) and 1 (unclassified *Veillonellaceae*) genera in the cecum and mid colon, respectively. Butyrate was found to be positively correlated (*p* < 0.05) with *Megasphaera* in the cecum and proximal colon. Iso-valerate was negatively correlated (*p* < 0.05) with 1, 2, and 3 genera and positively correlated (*p* < 0.05) with 4, 5, and 5 genera in the cecum, proximal colon, and mid colon, respectively. Valerate was positively correlated (*p* < 0.05) with *Megasphaera* and unclassified *Veillonellaceae* in the cecum and mid colon as well as *Megasphaera* and unclassified *Coriobacteriaceae* in the proximal colon. Valerate was also negatively correlated (*p* < 0.05) with five genera in the mid colon. Caproate was positively correlated (*p* < 0.05) with *Ruminococcus* in the cecum, *Desulfovibrio* in the proximal colon, and *Megasphaera* and *Succiniclasticum* in the mid colon. Caproate was also negatively correlated (*p* < 0.05) with unclassified *Veillonellaceae* in the proximal colon. Lactate was negatively correlated (*p* < 0.05) with 6 and 3 genera in the proximal and mid colon, respectively. Lactate was also positively correlated (*p* < 0.05) with *Megasphaera*, unclassified *Veillonellaceae*, and unclassified *Coriobacteriaceae* in the proximal colon and with *Megasphaera* in the mid colon.

**FIGURE 8:**
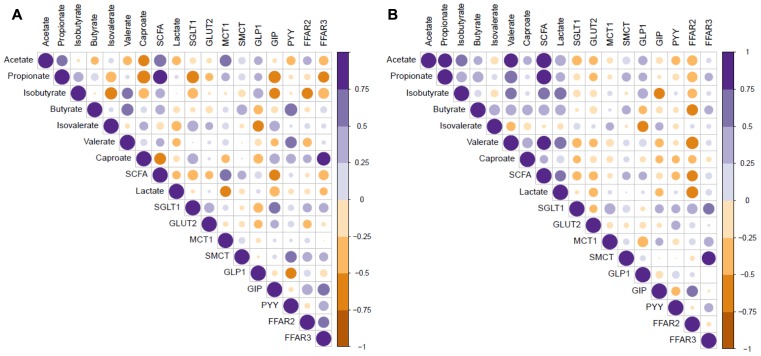
Correlation coefficients between microbial metabolites and mucosal gene expression in the cecum **(A)** and mid colon **(B)** of pigs fed control and transglycosylated starch diets. Ace, acetate; prop, propionate; but, butyrate; isobut, iso-butyrate; val, valerate; isoval, iso-valerate; cap, caproate; lac, lactate; SCFA, total short-chain fatty acids; CE, cecum; MC, mid colon.

Relationships between microbial metabolites and gene expression in gut mucosa in **Figure 8**. Expression of *SGLT1* was negatively correlated (*p* < 0.05) with propionate in the cecum (**Figure 8A**), whereas *GLP1* expression was negatively correlated (*p* < 0.05) with iso-valerate in both the cecum and mid colon. *FFAR2* expression was negatively correlated (*p* < 0.05) with iso-butyrate in the cecum and with butyrate, valerate, lactate, and total SCFA in the mid colon (**Figure 8B**).

## Discussion

Alterations in the gastrointestinal microbiota and fermentation patterns have been reported to mediate part of the RS-associated effects on host physiology (Birt et al., 2013; Yang et al., 2017). Consistent with this view, the present results demonstrated the capability of TGS to cause major shifts in bacterial community profiles throughout the gastrointestinal tract and stimulate hindgut fermentation. TGS-associated changes in the bacterial community were already apparent in gastric digesta, and largely differed from the effects of RS2 and RS3 on the intestinal microbiota (Sun et al., 2015, 2016; Umu et al., 2015; Venkataraman et al., 2016). Specifically, the TGS consistently enhanced an unclassified *Veillonellaceae* genus in ileal, cecal, proximal-colonic, and mid-colonic digesta, which appeared to replace other starch-degrading genera belonging to the *Ruminococcaceae* family. Present results further indicated a stronger effect of fermentation on intestinal gene expression than of the decreased glucose release in the small intestine at the time of sampling. The enhanced fermentation after TGS consumption in the hindgut may have up-regulated *GLP1* expression in the cecum and mid colon, thereby likely modifying insulin-dependent pathways (Bindels et al., 2013). The reduced glucose and energy availability from the TGS diet, and the subsequent reduced insulin signaling (Newman et al., 2017), in turn, likely contributed to the TGS-related decrease in adipocyte expression of genes related to lipid synthesis. Nevertheless, when interpreting the current data, it should be kept in mind that the sample size with eight animals per dietary treatment was small and that the study was conducted using young growing pigs of a specific age. Moreover, differences in gene expression levels are only the initial steps in adaptive processes and do not necessarily reflect functional protein patterns.

Compared to the large intestine relatively little information is available regarding RS-related changes, especially RS4, in bacterial profiles of the upper gastrointestinal segments. Although bacteria had access to other more easily fermentable substrates, such as the waxy cornstarch portion of the TGS diet, bacterial profile modifications by TGS were already apparent in gastric and ileal digesta. However, shifts in the gastric and ileal bacterial communities were less dramatic than those seen in the hindgut, which was supported by results from the α- and β-diversity analyses. Due to the relatively high availability of casein in the diet, which is an easily degradable protein source, *Proteobacteria* predominated in gastric digesta. Regardless, the TGS diet decreased the gastric abundance of the Gamma-Proteobacterium *Actinobacillus*. Some *Actinobacillus* species were reported to utilize starch (Sharma et al., 2014; Thuy et al., 2017) and therefore may have been replaced by genera from the *Veillonellaceae* (numerical increase of 9% with TGS) and *Coriobacteriaceae* families. These two genera were tentatively identified as *Selenomonas* and *Olsenella*, respectively; both include starch-degrading species (Kaneko et al., 2015; Li et al., 2015) and were even more drastically enriched by TGS in the hindgut segments. However, the depression in *Actinobacillus* was not apparent in the hindgut segments, indicating that their role in starch degradation in the present study primarily occurred in the stomach. In contrast, gastric and ileal abundances of starch-degrading *Ruminococcaceae* (Flint et al., 2008) was small and unaffected by the TGS diet in gastric and ileal digesta, emphasizing that their role in starch degradation was mainly in the hindgut. Most studies that found *Ruminococcus* as a significant genus for RS degradation investigated RS2 and RS3 (Martínez et al., 2010; Walker et al., 2011: Umu et al., 2015; Sun et al., 2016), whereas the majority of studies investigating other RS4 showed either no difference or a decreased relative abundance of *Ruminococcus* (Martínez et al., 2010: Metzler-Zebeli et al., 2015b), which is in agreement with present results. A possible rationale for this may be that, due to the α- and β-(1,2)- and (1,3)-linked glycosidic bonds present in our TGS product, hydrolysis of starch molecules by host enzymes was restricted, as reflected in the digestibility data. Moreover, it appears that the different glycosidic bonds also limited starch hydrolysis by certain bacterial amylases and pullulanases, which is supported by the reduced species richness and drastic shifts in the highly abundant genera in cecal, proximal-colonic, and mid-colonic digesta. However, we can only speculate about possible changes in the metabolic functions of the microbiota caused by the TGS as we did not complement the 16S rRNA sequencing with a function-based approach. Overall, the proximal colon showed the greatest bacterial changes, closely followed by the mid colon. This was reflected by the generally greater changes in SCFA concentrations and pH in the mid and proximal colon compared to the cecum, which suggests that microbial hydrolysis of these complex bonds in the TGS may have taken more time compared to the waxy cornstarch. In line with that, the retention time of digesta in the various gut segments and the nutrient availability may explain the greater effects observed in the stomach compared to the ileum.

Present digestibility values indicate that the TGS was not fully degraded in the gastrointestinal tract (4% more starch excretion in feces) compared to CON. This would allow for microbes that benefited from the TGS, either directly or through metabolic cross-feeding, to persist throughout the hindgut (Louis and Flint, 2017). Stimulation of metabolic cross-feeding among microbes with the TGS diet may be supported by the increased lactate concentrations especially in the proximal and mid colon. Microbial groups that appeared to benefit were mainly those belonging to the *Veillonellaceae* family (*Megasphaera*, *Succiniclasticum*, and *Mitsuokella*), which are capable of utilizing lactate and/or succinate and producing propionate, butyrate, and/or valerate (Soto-Cruz et al., 2002; Duncan et al., 2004; Tsukahara et al., 2006). Different networking among the dominant bacterial genera in pigs fed the CON diet compared to the TGS diet may have been supported by inverse correlations between the dominant bacterial genera in TGS-fed pigs [i.e., unclassified *Veillonellaceae* (tentatively identified as Selenomonas) and *Megasphaera*] and CON-fed pigs [i.e., *Ruminococcus* and unclassified *Ruminococcaceae* (tentatively identified as *Ruminococcus*)] with microbial metabolites (i.e., with lactate; Louis and Flint, 2017). Gastrointestinal pH, which was lower in TGS-fed pigs, may also have had a strong influence on competition between different bacterial groups in the microbial community, and therefore on the microbial metabolites produced (Louis and Flint, 2017). Furthermore, TGS-induced differences in proteolytic bacterial community or activity were indicated by increased iso-butyrate but decreased iso-valerate concentrations in the proximal and mid colon. As casein was the sole dietary protein source, microbe–microbe interactions, changes in intestinal passage rate and TGS-related changes in mucus production may explain these findings.

Although TGS modulated the gastric bacterial community and decreased starch digestibility, *SGLT1* expression in the small intestine, as the main route of mucosal glucose transport (Gorboulev et al., 2012), and serum glucose levels were similar between diets. Additionally, the jejunal and ileal incretin expression between diets indicated similar luminal glucose availability in the small intestine at the time point of sampling. As shown in our previous study with serial blood samplings, clear decreases in blood glucose and insulin were only discernible at very specific time points (30 and 210 min postprandially). Due to the nature of sample collection in a serial slaughter model, not all pigs were sampled within a 15–30 min time-frame. Therefore, it is not surprising that differences in blood glucose were not detected as reported in the companion paper (Newman et al., 2017). Overall, due to the high starch content in the CON diet, starch digestion may have been incomplete in the small intestine, leading to similar *SGLT1* expression levels between diets. Subsequently, a greater portion of the waxy cornstarch may have been available for degradation in the cecum (Moran et al., 2010), which could explain the lower expression of *SGLT1* in the cecal mucosa of TGS- compared to CON-fed pigs. Lower ileal substrate availability and the fact that *GLUT2* can translocate to the apical membrane to facilitate luminal glucose uptake in response to high dietary levels of rapidly digestible starch (Leturque et al., 2009) may have caused the down-regulated expression of *GLUT2* at the ileal mucosa of the TGS- compared to CON-fed pigs.

Despite TGS-related differences in lactate and SCFA concentrations in the cecum and colon, SCFA transporters *SMCT* and *MCT1* were not differentially expressed. Nevertheless, present correlations indicated a positive relationship between luminal SCFA (i.e., acetate) and MCT1 expression in the cecum. As simple diffusion is accelerated at lower pH values (Suzuki et al., 2008), it is possible that this transport route compensated for the increased SCFA and lactate concentrations in the colon of TGS-fed pigs, as large intestinal pH values were significantly lower in TGS compared to CON-fed pigs. After crossing the epithelium, acetate and propionate are transported through the portal vein to the liver, where propionate can induce intestinal gluconeogenesis (De Vadder et al., 2014). Therefore, the greater propionate concentrations in the cecum and proximal colon of TGS-fed pigs may have provided an additional energy source for these pigs compared to those fed the CON diet. This may be also supported by our recent findings of greater propionate levels in the peripheral blood of TGS- compared to CON-fed pigs (Newman et al., 2017).

Luminal SCFA, especially acetate, propionate, and butyrate, act as agonists of G protein-coupled receptors (e.g., FFAR-2 and FFAR-3) and can modify gut mucosal signaling with effects on appetite and energy homeostasis (McKenzie et al., 2017). Propionate mainly binds to FFAR-3, whereas acetate equally binds to FFAR-2 and FFAR-3 (McKenzie et al., 2017). From the SCFA data, it may be reasonable to assume an activation of FFAR-3 in the cecum and of FFAR-2 and FFAR-3 in mid colon of TGS-fed pigs. However, present results were contradictory, in that *FFAR2* and *FFAR3* tended to be less expressed in the cecum and were similarly expressed in the mid colon of TGS- compared to CON-fed pigs. These results were similar to those found in pigs fed an arabinoxylan-rich diet which had elevated levels of cecal SCFA (including acetate, propionate, and butyrate) and a down-regulated cecal *FFAR2* expression (Nielsen et al., 2014, 2015). Likewise, feeding RS3 to growing pigs elevated cecal and colonic SCFA concentrations, but showed similar mucosal *FFAR2* and *FFAR3* expression (Haenen et al., 2013). Despite the contradictory FFAR responses, *GLP1*, which is induced by intestinal SCFA (Tolhurst et al., 2012) via GPR signaling (e.g., FFAR-2 and FFAR-3; Chambers et al., 2015; Pais et al., 2016), was up-regulated in cecal and, to a lesser degree, mid-colonic mucosa in TGS-fed pigs.

The elevated expression of *GLP1* in TGS-fed pigs likely modulated insulin secretion (Bindels et al., 2013), which together with the decreased serum insulin observed in our previous TGS-fed pigs (Newman et al., 2017) and the lower energy digestibility of the TGS diet, likely contributed to the down-regulated expression of genes related to fatty acid synthesis in adipose tissue. The present effects on lipid metabolism are in general agreement with previous studies investigating the effects of CMS, which have shown numerous effects on lipid metabolism in healthy humans, mice, and pigs (Shimotoyodome et al., 2010, 2011; Metzler-Zebeli et al., 2015a; Newman et al., 2017). Genes involved in fatty acid synthesis were conversely regulated by dietary TGS consumption in adipose tissue as compared to the liver, which is in agreement with previous findings showing that fat synthesis in adipose and hepatic tissues in pigs may be differentially regulated (Duran-Montgé et al., 2008). Unlike in humans and rodents, *de novo* lipid synthesis in pigs primarily occurs in adipose tissue (O’Hea and Leveille, 1969). Since insulin is needed for adipocytic triglyceride storage (Dimitriadis et al., 2011), the TGS-induced decrease in postprandial insulin secretion likely explains the down-regulation of fatty acid and cholesterol synthesis genes in adipocytes and hence the utilization of serum lipids for peripheral lipid synthesis in TGS-fed pigs, whereas in hepatocytes nutrients deriving from the portal vein may have induced the expression of genes for lipogenesis. Although not measured in the present study, the TGS-related reduction in insulin was probably accompanied by increased postprandial levels of glucagon, thereby decreasing the insulin:glucagon ratio and further diminishing the effect of insulin signaling. Haenen et al. (2013), for example, showed enhanced cecal expression of glucagon after RS3 consumption. Gene expression data support up- and down-regulation of enzymes in *de novo* lipogenesis via nuclear transcription factor SREBP1 in hepatic and adipose tissues, respectively, and via down-regulation of *FFAR2* and *FFAR3* expression in adipose tissue. By contrast, the expression of *CPT1*, which is involved in fatty acid oxidation (Warfel et al., 2017), remained unaffected by dietary treatment. Moreover, in contrast to our assumption, the TGS did not appear to increase satiety in our pigs as the feed intake between TGS- and CON-fed pigs was similar. This may be supported by similar intestinal expression levels of the appetite-suppressing hormone *PYY* between feeding groups. In addition, the down-related expression level of the satiety hormone *LEP*, likely a consequence of decreased storage of lipids, in adipocytes of TGS-fed compared to CON-fed pigs may have modulated the feed intake behavior of our pigs (Polyzos et al., 2015).

## Conclusion

The consumption of the TGS diet altered the most abundant bacterial genera in the stomach, cecum, proximal colon, and mid colon as well as caused greater microbial metabolite concentrations in the hindgut compared to the CON diet. Greater relative abundances of an unclassified *Veillonellaceae* genus, *Megasphaera, Mitsuokella*, and *Succiniclasticum* – which contain many propionate-producing species – appeared to replace other starch-degrading genera belonging to the *Ruminococcaceae* family. Many TGS-associated changes in the bacterial community were already apparent in gastric and ileal digesta. Consumption of the TGS diet enhanced propionate fermentation in the cecum and proximal colon, which may have induced the up-regulated expression of *GLP1* in the cecum and mid colon. However, genes involved in fatty acid synthesis (*FASN, SREBP1*, and *ACACA*) were conversely regulated in adipocytes as compared to hepatocytes in TGS-fed pigs. This was likely associated with the decreased starch digestibility of the TGS diet, which reduced insulin signaling and hence contributed to the down-regulated expression of lipid synthesis genes.

## Author Contributions

Conceived and designed the experiments: BM-Z, QZ, and DG. Performed the experiments: BM-Z. Analyzed the data: MN, RP, and BM-Z. Interpreted the data: MN. Drafted the manuscript: MN and BM-Z. Revised the manuscript: RP, QZ, and DG. Primary responsibility for the final content: BM-Z. All of the authors read and approved the final manuscript.

## Conflict of Interest Statement

Agrana Research & Innovation Center GmbH provided support in the form of salaries for author DG. The other authors declare that the research was conducted in the absence of any commercial or financial relationships that could be construed as a potential conflict of interest.

## Footnotes

1 www.ncbi.nlm.nih.gov/tools/primer-blast/

2 http://greengenes.lbl.gov
