# Supplementary material for: Transglycosylated Starch Modulates the Gut Microbiome and Expression of Genes Related to Lipid Synthesis in Liver and Adipose Tissue of Pigs
Source: Front Microbiol. 2018 Feb 13;9:224. doi: 10.3389/fmicb.2018.00224 (PMC5816791; doi:10.3389/fmicb.2018.00224)
Supplement: Supplementary file 1 [file Data_Sheet_1.DOCX]

Supplementary Material

Transglycosylated Starch Alters Gut Microbiome and Inversely Affects Gene Expression Related to Lipid Synthesis in Liver and Adipose Tissue of Pigs

Monica A. Newman^1^, Renée M. Petri^1^, Dietmar Grüll^2^, Qendrim Zebeli^1^, Barbara U. Metzler-Zebeli^1^*

*** Correspondence:** Dr. Barbara Metzler-Zebeli: barbara.metzler@vetmeduni.ac.at

# Supplementary Figure and Tables

## Supplementary Figure


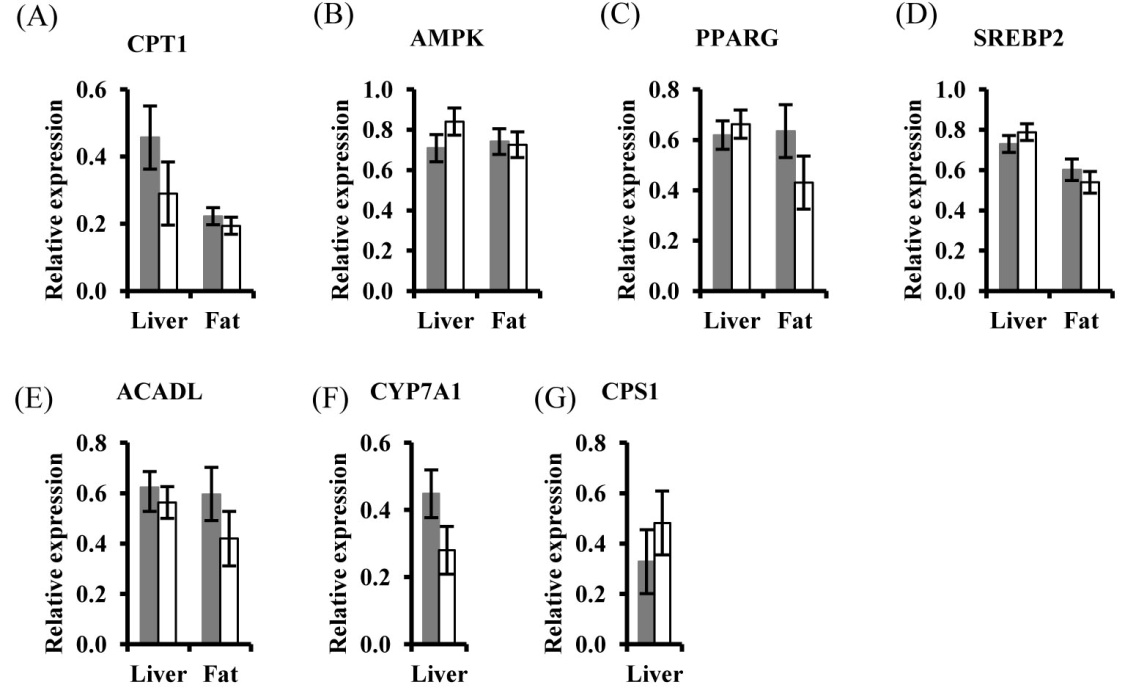


**Figure S1.** Relative expression of target genes in liver and abdominal fat samples that did not significantly differ between pigs fed the control (■) or transglycosylated starch (□) diet. Relative (A) *CPT1*, (B) *AMPK*, (C) *PPARG*, (D) *SREBP2*, (E) *ACADL*, (F) *CYP7A1*, and (G) *CPS1* expression. Values are presented as least square means ± SEM; *n* = 8 per dietary treatment.

## Supplementary Tables

**Table S1.** Ingredient and analyzed nutrient composition of experimental diets

| **Item** | **CON diet^1^** | **TGS** **diet**^2^ |
| --- | --- | --- |
| **Ingredient composition, %** | | |
| Waxy cornstarch | 72.1 | 36.05 |
| Transglycosylated cornstarch | 0.00 | 36.05 |
| Casein | 18.00 | 18.00 |
| Lignocellulose^3^ | 4.00 | 4.00 |
| Rapeseed oil | 1.00 | 1.00 |
| Monocalcium phosphate | 4.00 | 4.00 |
| Vitamin-mineral premix^4^ | 0.60 | 0.60 |
| Titanium dioxide | 0.30 | 0.30 |
| **Analyzed nutrient composition, g/kg** | | |
| Gross energy (MJ/kg) | 16.52 | 16.66 |
| Dry matter | 933 | 942 |
| Crude protein | 165 | 169 |
| Total starch | 728 | 709 |
| Calcium | 9.4 | 9.5 |
| Phosphorus | 5.7 | 5.7 |

Values are presented on a dry matter basis. ^1^CON, control starch; ^2^TGS, transglycosylated cornstarch (ARIC, Tulln, Austria). ^3^FibreCell (agromed Austria GmbH, Austria); ^4^Provided per kilogram of complete diet (GARANT GmbH, Austria): 16,000 IU of vitamin A, 2,000 IU of vitamin D_3_, 125 mg of vitamin E, 2.0 mg of vitamin B_1_, 6.0 mg of vitamin B_2_, 3.0 mg of vitamin B_6_, 0.03 mg of vitamin B_12_, 3.0 mg of vitamin K_3_, 30 mg of niacin, 15.0 mg of pantothenic acid, 900 mg of choline chloride, 0.15 mg of biotin, 1.5 mg of folic acid, 200 mg of vitamin C; 4.6 g of Ca, 2.3 g as digestible P, 2.4 g as Na, 2.0 g of Cl, 3.2 g K, 1.0 g Mg; 50 mg of Mn (as MnO); 100 mg of Zn (as ZnSO_4_); 120 mg of Fe (as FeSO_4_), 15.6 mg of Cu (as CuSO_4_), 0.5 mg of Se (as Na_2_SeO_3_), 1.9 mg of I (as Ca(IO_3_)_2_).

| **Table S2.** Forward (F) and reverse (R) primers for quantitative PCR, PCR efficiency, and coefficient correlation of standard curves. | | | | | | | |
| --- | --- | --- | --- | --- | --- | --- | --- |
| Gene | Complete gene name | Accession number | Oligonucleotide sequence (5’ to 3’) | Amplicon size (bp) | PCR efficiency (%) | *R*^2^ | Reference |
| *SGLT1* | Sodium/Glucose Cotransporter 1 | NM_001164021.1 | F: TGTCTTCCTCATGGTGCCAA  R: AGGAGGGTCTCAGGCCAAA | 149 | 108.0 | 0.99 | 1 |
| *GLUT2* | Facilitated Glucose Transporter 2 | NM_001097417.1 | F: TACGGCATCTGCTAGCCTCAT  R: CCACCAATTGCAAAGATGGAC | 66 | 89.3 | 1.00 | 1 |
| *MCT1* | Monocarboxylate transporter 1 | AM286425.1 | F: GGTGGAGGTCCTATCAGCAG  R: AAGCAGCCGCCAATAATCAT | 74 | 96.4 | 0.99 | 1 |
| *SMCT* | Sodium-coupled monocarboxylate transporter | XM_003122908.1 | F: AGGTCTACCGCTTTGGAGCAT  R: GAGCTCTGATGTGAAGATGATGACA | 77 | 82.3 | 0.99 | 1 |
| *GLP1* | Glucagon-like peptide 1 | NM_001256594.1 | F: GCTGATGGTGGCGATCTTGT  R: TCCCAGCTCTTCCGAAACTC | 69 | 98.1 | 0.99 | 2 |
| *GIP* | Gastric inhibitory polypeptide | NM_001287408.1 | F: GGATGGTGGAGCAGTTGGA  R: CCAATCCTGAGCTGGGTTTG | 71 | 88.1 | 0.99 | 2 |
| *PYY* | Peptide tyrosine tyrosine | AY344365.1 | F: AGATATGCTAATACACCGAT  R: CCAAACCCTTCTCAGATG | 94 | 98.5 | 0.99 | 9 |
| *FFAR2* | Free fatty acid receptor 2 | NM_001278758.1 | F: CTGCCTGGGATCGTCTGTG  R: CATACCCTCGGCCTTCTGG | 249 | 104.5 | 0.99 | 10 |
| *FFAR3* | Free fatty acid receptor 3 | NM_001315601.1 | F:GCCCTTGCCCTTCATCTTCT R:CCGGGTCTTGTACCAGAGTG | 136 | 103.8 | 0.99 | Newly designed |
| *CPT1* | Carnitine palmitoyltransferase 1A | NM_001129805.1 | F: GACGAGGACCCCTGATGGTG  R: CGTGGATCCCAGGAGAATCG | 167 | 91.5 | 0.99 | 8 |
| *AMPK* | AMP-Activated protein kinase Subunit Alpha 2 | NM_214266.1 | F: GCTCCTTGACCGGAAGCA  R: CAGACTGGCACACATTTCGAA | 86 | 105.1 | 0.99 | 3 |
| *FASN* | Fatty acid synthase | NM_001099930.1 | F: CGGTCTTGCTGACCAAGAAG  R: TTGGAACCGTCTGTGTTCGT | 82 | 97.5 | 0.99 | 3 |
| *CYP7A1* | Cholesterol 7 alpha-hydroxylase | NM_001005352.3 | F: TTCCCGATTCATGTGTTCAA  R: ACCAGTTCCGAGATGTGGTC | 104 | 95.7 | 0.99 | 6 |
| *HMGCR* | 3-hydroxy-3-methylglutaryl-CoA reductase | NM_001122988.1 | F: CTGCACCATGCCATCCATAG  R: CTTTGCACGCTCCTTGAACC | 104 | 99.9 | 0.99 | 7 |
| *PPARG* | Peroxisome proliferator-activated receptor γ | NM_214379.1 | F: CATTCCCGAGAGCTGATCC  R: GGAAGGCTCTTCGTGAGGTT | 149 | 110.4 | 0.99 | 3 |
| *SREBP1* | Sterol regulatory element-binding protein 1 | NM_214157.1 | F: TCCATCAATGACAAGATCATCGA  R: CTGGTTGCTCTGCTGAAGGAA | 123 | 86.4 | 1.00 | 3 |
| *SREBP2* | Sterol regulatory element-binding protein 2 | DQ020476.1 | F: GCTTCTCCCCCTACTCCATC  R: GAGAGGCACAGGAAGGTGAG | 151 | 94.2 | 0.99 | 4 |
| *ACACA* | Acetyl-CoA carboxylase alpha | NM_001114269.1 | F: GGCCATCAAGGACTTCAACC  R. ACGATGTAAGCGCCGAACTT | 120 | 92.7 | 0.99 | 5 |
| *ACADL* | Acyl-CoA dehydrogenase - long chain | NM_213897.1 | F: GTAAGAACAAATGCCAAGA  R: CAGCCACTACAATCACAAC | 103 | 94.0 | 0.99 | 11 |
| *CPS* | Carbamoyl-phosphate synthase 1 | XM_005672159.2 | F: ATCCAGCACCCGTATCTATGC  R: GAGTGAATCAGAGCACTGGG | 178 | 98.3 | 0.99 | Newly designed |
| *LEP* | Leptin | NM_213840.1 | F:TGGAAGCCTCCCTCTACTCC  R:CTCAGGTTTCTTCCCCCGAC | 146 | 101.2 | 0.99 | Newly designed |
| **Housekeeping genes** | | | | | | | |
| *ACTB* | Actin | XM_003357928.1 | F: GGGCATCCTGACCCTCAAG  R: TGTAGAAGGTGTGATGCCAGATCT | 89 | 97.3 | 0.99 | 2 |
| *GAPDH* | Glyceraldehyde-3-phosphate dehydrogenase | NM_001206359.1 | F: GGCGTGAACCATGAGAAGTATG  R: GGTGCAGGAGGCATTGCT | 60 | 96.5 | 0.99 | 2 |
| *B2M* | Beta-2-microglobulin | NM_213978.1 | F: CCCCCGAAGGTTCAGGTT  R: GCAGTTCAGGTAATTTGGCTTTC | 66 | 102.2 | 0.99 | 2 |
| *HPRT* | Hypoxanthine phosphoribosyltransferase | NM_001032376.2 | F: AGAAAAGTAAGCAGTCAGTTTCATATCAGT  R: ATCTGAACAAGAGAGAAAATACAGTCAATAG | 131 | 92.1 | 0.99 | 2 |
| *OAZ1* | Ornithine decarboxylase antizyme 1 | NM_001122994.1 | F: TCGGCTGAATGTAACAGAGGAA  R: GAGCCTGGATTGGACGTTTAAA | 70 | 99.2 | 0.99 | 2 |

References

1. Metzler-Zebeli B.U., Mann E., Ertl R., Schmitz-Esser S., Wagner M., Klein D., Ritzmann M., Zebeli Q. (2015). Dietary calcium concentration and cereals differentially affect mineral balance and tight junction proteins expression in jejunum of weaned pigs. Br. J. Nutr. 113, 1019-1031. 10.1017/S0007114515000380

2. Metzler-Zebeli B.U., Ertl R., Grüll D., Molnar T., Zebeli Q. (2016). Enzymatically modified starch up-regulates expression of incretins and sodium-coupled monocarboxylate transporter in jejunum of growing pigs. Animal. 1-9. 10.1017/S1751731116002615

3. Metzler-Zebeli B.U., Ertl R., Klein D., Zebeli Q. (2015). Explorative study of metabolic adaptations to various dietary calcium intakes and cereal sources on serum metabolome and hepatic gene expression in juvenile pigs. Metabolomics. 11, 545-558. 10.1007/s11306-014-0714-2

4. Rideout T.C., Yuan Z., Bakovic M., Liu Q., Li R., Mine Y., Fan M.Z. (2007). Guar gum consumption increases hepatic nuclear SREBP2 and LDL receptor expression in pigs fed an atherogenic diet. J. Nutr. 137, 568-572.

5. Madeira M.S., Pires V.M.R., Alfaia C.M., Costa A.S.H., Luxton R., Doran O., Bessa R.J.B., Prates J.A.M. (2013). Differential effects of reduced protein diets on fatty acid composition and gene expression in muscle and subcutaneous adipose tissue of Alentejana purebred and Large White × Landrace × Peitrain crossbred pigs. Br. J. Nutr. 110, 216-229. 10.1017/S0007114512004916

6. Gunawan A., Sahadevan S., Neuhoff C., Grosse-Brinkhaus C., Gad A., Frieden L., Tesfaye D., Tholen E., Looft C., Uddin M.J., Schellander K., Cinar M.U. (2013). RNA deep sequencing reveals novel candidate genes and polymorphisms in boar testis and liver tissues with divergent androstenone levels. PLoSONE. 8, e63259. 10.1371/journal.pone.0063259

7. Oster, M., Muráni E., Ponsuksili S., D’Eath R.B., Turner S.P., Evans G., Thölking L., Kurt E., Klont R., Foury A., Mormede P., Wimmers K. (2014). Hepatic expression patterns in psychosocially high-stressed pigs suggest mechanisms following allostatic principles. Physiol. Behav. 128, 159-165. 10.1016/j.physbeh.2014.02.014

8. Polakof S., Rémond D., Rambeau M., Pujos-Guillot E., Sébédio J., Dardevet D., Comte B., Savary-Auzeloux I. (2015). Metabolomics. 11, 964-979. 10.1007/s11306-014-0753-8

9. Haenen D., Zhang J., Souza da Silva C., Bosch G., van der Meer I.M., van Arkel J., van den Borne J.J.G.C., Gutiérrez O.P., Smidt H., Kemp B., Müller M., Hooiveld G.J.E.J. (2013). A diet high in resistant starch modulates microbiota composition, SCFA concentrations, and gene expression in pig intestine. J. Nutr. 143, 274-283. 10.3945/jn.112.169672

10. Li G., Su H., Zhou Z., Yao W. (2014). Identification of the porcine G protein-coupled receptor 41 and 43 genes and their expression pattern in different tissues and development stages. PLoS ONE. 9, e97342. 10.1371/journal.pone.0097342

11. Zhou C., Zhang J., Ma J., Jiang A., Tang G., Mai M., Zhu L., Bai L., Li M., Li X. (2013). Gene expression profiling reveals distinct features of various porcine adipose tissues. Lipids Health Dis. 12, 1-13. 10.1186/1476-511X-12-75

| **Table S3.** Performance measurements and nutrient digestibility of pigs fed transglycosylated (TGS) or control (CON) starch diets^1^ | | | | |
| --- | --- | --- | --- | --- |
| **Item, %** | **CON** | **TGS** | **SEM** | ***P*-value** |
| **Performance measurements** |  |  |  |  |
| Average daily feed intake, kg DM | 1.70 | 1.61 | 0.093 | 0.366 |
| Average daily gain, kg | 1.04 | 1.10 | 0.077 | 0.433 |
| Feed:Gain | 1.63 | 1.49 | 0.082 | 0.097 |
| **Apparent total tract digestibility, %** |  |  |  |  |
| Dry matter | 93.9 | 88.3 | 0.44 | <0.001 |
| Gross energy | 93.2 | 86.3 | 0.49 | <0.001 |
| Starch | 99.9 | 95.8 | 0.43 | <0.001 |
| Crude protein | 95.5 | 90.4 | 0.39 | <0.001 |
| Crude ash | 70.8 | 71.5 | 1.96 | 0.727 |
| Calcium | 69.8 | 66.3 | 3.60 | 0.238 |
| Phosphorus | 73.6 | 70.2 | 1.86 | 0.223 |

^1^Data are presented as least square means ± SEM; *n* = 8 per dietary treatment.

| **Table S4.** Blood serum parameters and jejunal brush border enzymes from pigs fed transglycosylated (TGS) or control (CON) starch diets | | | | |
| --- | --- | --- | --- | --- |
| **Item** | **CON** | **TGS** | **SEM** | ***P*-value** |
| **Brush border enzymes** |  |  |  |  |
| Lactase, U/g | 83 | 58 | 13.4 | 0.084 |
| Maltase, U/g | 347 | 362 | 27.3 | 0.588 |
| Sucrase, U/g | 29 | 32 | 5.5 | 0.648 |
| **Blood serum parameters** |  |  |  |  |
| Haptoglobin, g/L | 1.52 | 1.16 | 0.282 | 0.227 |
| Glucose, mmol/L | 7.88 | 7.98 | 0.884 | 0.914 |
| Urea, mmol/L | 4.88 | 6.03 | 0.904 | 0.225 |
| Cholesterol, mmol/L | 1.68 | 1.63 | 0.096 | 0.599 |
| Triglycerides, mmol/L | 0.24 | 0.21 | 0.047 | 0.540 |
| NEFA, mmol/L | 0.12 | 0.10 | 0.012 | 0.257 |

^1^Data are presented as least square means ± SEM; *n* = 8 per dietary treatment. NEFA = non-esterified fatty acids

| **Table S5.** The 30 most abundant genera in gastrointestinal digesta of pigs fed transglycosylated (TGS) or control (CON) starch diets^1^ | | | | | | | | |
| --- | --- | --- | --- | --- | --- | --- | --- | --- |
|  | | **CON** | | | **TGS** | | **SEM** | ***P*-value** |
| **Gastric digesta, %** | |  | | |  | |  |  |
| Unclassified *Enterobacteriaceae* | | 35.04 | | | 37.43 | | 7.132 | 0.816 |
| *Actinobacillus* | | 26.09 | | | 11.02 | | 3.630 | 0.011 |
| *Acinetobacter* | | 9.54 | | | 6.99 | | 1.940 | 0.153 |
| Unclassified *Veillonellaceae* | | 2.65 | | | 11.10 | | 4.581 | 0.109 |
| *Moraxella* | | 3.59 | | | 5.35 | | 2.300 | 0.391 |
| *Eikenella* | | 3.32 | | | 4.73 | | 1.499 | 0.517 |
| Unclassified *Neisseriaceae* | | 4.00 | | | 3.45 | | 1.338 | 0.737 |
| *Haemophilus* | | 1.67 | | | 2.17 | | 0.719 | 0.452 |
| *Ruminococcus* | | 0.18 | | | 2.27 | | 1.063 | 0.152 |
| *Streptococcus* | | 1.20 | | | 1.22 | | 0.273 | 0.968 |
| *Megasphaera* | | 0.30 | | | 2.09 | | 0.734 | 0.064 |
| *Pseudomonas* | | 1.83 | | | 0.46 | | 0.667 | 0.169 |
| Unclassified *Aeromonadaceae* | | 2.09 | | | 0.02 | | 1.388 | 0.310 |
| *Leptotrichia* | | 0.80 | | | 1.28 | | 0.416 | 0.427 |
| *Mitsuokella* | | 0.21 | | | 1.21 | | 0.545 | 0.219 |
| Unclassified *Coriobacteriaceae* | | 0.05 | | | 1.15 | | 0.290 | 0.018 |
| *Aggregatibacter* | | 0.76 | | | 0.41 | | 0.142 | 0.101 |
| *Lactobacillus* | | 0.31 | | | 0.66 | | 0.109 | 0.036 |
| Unclassified *Mitochondria* | | 0.38 | | | 0.28 | | 0.152 | 0.647 |
| Unclassified *Pasteurellaceae* | | 0.36 | | | 0.24 | | 0.128 | 0.406 |
| *Turicibacter* | | 0.24 | | | 0.35 | | 0.111 | 0.464 |
| *Succiniclasticum* | | 0.04 | | | 0.54 | | 0.126 | 0.014 |
| *Collinsella* | | 0.24 | | | 0.34 | | 0.217 | 0.668 |
| *Klebsiella* | | 0.40 | | | 0.18 | | 0.206 | 0.457 |
| Unclassified *Microbacteriaceae* | | 0.35 | | | 0.19 | | 0.085 | 0.069 |
| Unclassified *Christensenellaceae* | | 0.21 | | | 0.27 | | 0.098 | 0.647 |
| Unclassified *Moraxellaceae* | | 0.18 | | | 0.28 | | 0.058 | 0.276 |
| *Mannheimia* | | 0.33 | | | 0.12 | | 0.112 | 0.070 |
| Unclassified *Gemellaceae* | | 0.12 | | | 0.33 | | 0.088 | 0.089 |
| Unclassified *Ruminococcaceae* | | 0.24 | | | 0.19 | | 0.139 | 0.595 |
| **Ileal digesta, %** | |  | | |  | |  |  |
| *Turicibacter* | | 43.34 | | | 54.33 | | 12.472 | 0.553 |
| Unclassified *Enterobacteriaceae* | | 32.28 | | | 29.89 | | 12.436 | 0.893 |
| Unclassified *Clostridiaceae* | | 16.58 | | | 6.54 | | 10.154 | 0.492 |
| *Actinobacillus* | | 0.75 | | | 4.46 | | 2.429 | 0.213 |
| Unclassified *Aeromonadaceae* | | 4.28 | | | 0.003 | | 4.203 | 0.467 |
| *Campylobacter* | | 0.04 | | | 1.41 | | 0.708 | 0.203 |
| *Klebsiella* | | 0.77 | | | 0.001 | | 0.690 | 0.453 |
| *Clostridium* | | 0.33 | | | 0.27 | | 0.271 | 0.837 |
| Unclassified *Clostridiales* | | 0.19 | | | 0.38 | | 0.104 | 0.073 |
| *Helicobacter* | | 0.08 | | | 0.38 | | 0.150 | 0.188 |
| *Streptococcus* | | 0.32 | | | 0.13 | | 0.261 | 0.505 |
| Unclassified *Helicobacteraceae* | | 0.06 | | | 0.27 | | 0.105 | 0.180 |
| *Eikenella* | | 0.02 | | | 0.28 | | 0.144 | 0.219 |
| *Aggregatibacter* | | 0.03 | | | 0.20 | | 0.071 | 0.025 |
| Unclassified *Veillonellaceae* | | 0.03 | | | 0.16 | | 0.047 | 0.039 |
| *Haemophilus* | | 0.003 | | | 0.15 | | 0.076 | 0.156 |
| *Pseudomonas* | | 0.14 | | | 0.002 | | 0.133 | 0.450 |
| *Ruminococcus* | | 0.07 | | | 0.06 | | 0.040 | 0.823 |
| *Megasphaera* | | 0.02 | | | 0.12 | | 0.008 | <0.001 |
| *Flexispira* | | 0.02 | | | 0.11 | | 0.038 | 0.150 |
| *Mitsuokella* | | 0.01 | | | 0.11 | | 0.027 | 0.027 |
| *Acinetobacter* | | 0.09 | | | 0.02 | | 0.050 | 0.327 |
| *Salmonella* | | 0.09 | | | 0.00 | | 0.051 | 0.266 |
| Unclassified *Neisseriaceae* | | 0.02 | | | 0.06 | | 0.032 | 0.328 |
| Unclassified *Ruminococcaceae* | | 0.04 | | | 0.03 | | 0.007 | 0.243 |
| *Lactobacillus* | | 0.02 | | | 0.05 | | 0.024 | 0.488 |
| *Collinsella* | | 0.00 | | | 0.07 | | 0.032 | 0.187 |
| Unclassified *Christensenellaceae* | | 0.04 | | | 0.02 | | 0.010 | 0.243 |
| *Selenomonas* | | 0.003 | | | 0.04 | | 0.020 | 0.209 |
| Unclassified *Comamonadaceae* | | 0.01 | | | 0.03 | | 0.013 | 0.208 |
| **Cecal digesta, %** | |  | | |  | |  |  |
| Unclassified *Veillonellaceae* | | 0.90 | | | 37.56 | | 6.740 | 0.002 |
| *Ruminococcus* | | 30.40 | | | 3.33 | | 8.880 | 0.014 |
| *Anaerovibrio* | | 10.76 | | | 10.83 | | 7.049 | 0.995 |
| Unclassified *Clostridiales* | | 12.69 | | | 6.28 | | 7.177 | 0.396 |
| Unclassified *Enterobacteriaceae* | | 4.96 | | | 8.48 | | 5.738 | 0.362 |
| *Ruminobacter* | | 8.38 | | | 0.07 | | 4.862 | 0.199 |
| *Succinivibrio* | | 7.27 | | | 1.13 | | 4.663 | 0.304 |
| *Megasphaera* | | 0.63 | | | 7.52 | | 1.799 | 0.017 |
| Unclassified *Ruminococcaceae* | | 6.54 | | | 1.47 | | 1.751 | 0.023 |
| Unclassified *RF39* | | 3.73 | | | 3.51 | | 4.278 | 0.963 |
| *Turicibacter* | | 1.29 | | | 4.95 | | 1.955 | 0.207 |
| *Mitsuokella* | | 0.24 | | | 5.21 | | 2.399 | 0.086 |
| *Akkermansia* | | 2.52 | | | 0.12 | | 0.881 | 0.075 |
| Unclassified *RF32* | | 0.15 | | | 2.21 | | 1.552 | 0.364 |
| *Succiniclasticum* | | 0.07 | | | 2.13 | | 0.785 | 0.058 |
| Unclassified Bacteroidales | | 1.70 | | | 0.25 | | 0.612 | 0.040 |
| Unclassified Clostridiaceae | | 1.15 | | | 0.29 | | 0.455 | 0.114 |
| *Helicobacter* | | 0.53 | | | 0.91 | | 0.663 | 0.570 |
| Unclassified *Christensenellaceae* | | 0.90 | | | 0.32 | | 0.219 | 0.079 |
| Unclassified *Tremblayales* | | 1.07 | | | 0.15 | | 0.495 | 0.209 |
| *Phascolarctobacterium* | | 0.55 | | | 0.56 | | 0.251 | 0.980 |
| Unclassified *Lachnospiraceae* | | 0.61 | | | 0.27 | | 0.325 | 0.480 |
| *Desulfovibrio* | | 0.46 | | | 0.30 | | 0.133 | 0.392 |
| *Sutterella* | | 0.20 | | | 0.36 | | 0.165 | 0.495 |
| *Oscillospira* | | 0.28 | | | 0.09 | | 0.068 | 0.074 |
| Unclassified *Succinivibrionaceae* | | 0.21 | | | 0.15 | | 0.212 | 0.796 |
| Unclassified *Desulfovibrionaceae* | | 0.25 | | | 0.09 | | 0.097 | 0.084 |
| Unclassified *Coriobacteriaceae* | | 0.02 | | | 0.31 | | 0.083 | 0.029 |
| *Collinsella* | | 0.08 | | | 0.15 | | 0.121 | 0.609 |
| *Actinobacillus* | | 0.02 | | | 0.20 | | 0.083 | 0.145 |
| **Proximal-colonic digeta, %** | |  | | |  | |  |  |
| Unclassified *Veillonellaceae* | 1.54 | | | 52.09 | | 6.728 | | 0.000 |
| *Ruminococcus* | 27.46 | | | 2.56 | | 6.022 | | 0.005 |
| *Anaerovibrio* | 10.17 | | | 6.89 | | 6.441 | | 0.724 |
| Unclassified *Enterobacteriaceae* | 6.18 | | | 8.57 | | 6.039 | | 0.721 |
| Unclassified *Clostridiales* | 11.35 | | | 1.02 | | 4.036 | | 0.014 |
| Unclassified *Ruminococcaceae* | 9.96 | | | 1.60 | | 2.061 | | 0.013 |
| *Ruminobacter* | 10.57 | | | 0.03 | | 6.321 | | 0.223 |
| *Megasphaera* | 1.06 | | | 6.58 | | 1.195 | | 0.006 |
| *Turicibacter* | 1.26 | | | 4.02 | | 1.263 | | 0.145 |
| *Succinivibrio* | 3.54 | | | 0.24 | | 1.336 | | 0.085 |
| *Mitsuokella* | 0.17 | | | 3.45 | | 1.241 | | 0.012 |
| *Succiniclasticum* | 0.06 | | | 3.27 | | 1.300 | | 0.019 |
| Unclassified *RF39* | 2.93 | | | 0.05 | | 1.971 | | 0.317 |
| Unclassified *Christensenellaceae* | 1.71 | | | 0.72 | | 0.427 | | 0.121 |
| *Helicobacter* | 0.63 | | | 1.75 | | 0.472 | | 0.113 |
| *Akkermansia* | 1.71 | | | 0.02 | | 0.608 | | 0.069 |
| Unclassified *Bacteroidales* | 1.54 | | | 0.09 | | 0.391 | | 0.015 |
| Unclassified *Lachnospiraceae* | 0.63 | | | 0.98 | | 0.539 | | 0.652 |
| Unclassified *Coriobacteriaceae* | 0.05 | | | 1.17 | | 0.232 | | 0.004 |
| *Desulfovibrio* | 0.83 | | | 0.38 | | 0.101 | | 0.007 |
| Unclassified *Clostridiaceae* | 1.10 | | | 0.06 | | 0.283 | | 0.013 |
| *Sutterella* | 0.61 | | | 0.40 | | 0.225 | | 0.524 |
| *RFN20* | 0.05 | | | 0.88 | | 0.383 | | 0.144 |
| *Phascolarctobacterium* | 0.58 | | | 0.29 | | 0.114 | | 0.098 |
| *Campylobacter* | 0.12 | | | 0.47 | | 0.237 | | 0.143 |
| Unclassified *RF32* | 0.34 | | | 0.24 | | 0.182 | | 0.711 |
| Unclassified *Tremblayales* | 0.39 | | | 0.17 | | 0.167 | | 0.363 |
| Unclassified *Alphaproteobacteria* | 0.47 | | | 0.01 | | 0.213 | | 0.145 |
| Unclassified *Desulfovibrionaceae* | 0.39 | | | 0.08 | | 0.108 | | 0.005 |
| *Collinsella* | 0.10 | | | 0.37 | | 0.145 | | 0.198 |
| **Mid-colonic digesta, %** |  | | |  | |  | |  |
| Unclassified *Veillonellaceae* | | | 9.16 | 51.63 | | 9.241 | | 0.006 |
| *Ruminococcus* | | | 31.11 | 3.59 | | 5.986 | | 0.006 |
| Unclassified *Ruminococcaceae* | | | 14.61 | 2.58 | | 2.386 | | 0.003 |
| Unclassified *Clostridiales* | | | 12.74 | 1.02 | | 4.693 | | 0.031 |
| Unclassified *Enterobacteriaceae* | | | 3.28 | 7.62 | | 5.200 | | 0.328 |
| *Megasphaera* | | | 1.50 | 6.83 | | 1.563 | | 0.007 |
| *Anaerovibrio* | | | 2.87 | 4.76 | | 1.944 | | 0.504 |
| *Succinivibrio* | | | 6.35 | 0.24 | | 3.532 | | 0.206 |
| Unclassified *Christensenellaceae* | | | 3.69 | 1.14 | | 1.075 | | 0.115 |
| *Helicobacter* | | | 0.39 | 4.08 | | 1.788 | | 0.167 |
| *Mitsuokella* | | | 0.18 | 3.84 | | 1.837 | | 0.145 |
| *Succiniclasticum* | | | 0.13 | 3.63 | | 1.225 | | 0.038 |
| *Ruminobacter* | | | 3.30 | 0.01 | | 2.187 | | 0.293 |
| Unclassified *RF39* | | | 3.00 | 0.15 | | 1.791 | | 0.279 |
| Unclassified *Coriobacteriaceae* | | | 0.06 | 2.00 | | 0.605 | | 0.003 |
| *Turicibacter* | | | 0.49 | 1.37 | | 0.438 | | 0.181 |
| Unclassified *Bacteroidales* | | | 1.49 | 0.06 | | 0.296 | | 0.004 |
| *RFN20* | | | 0.06 | 1.38 | | 0.575 | | 0.127 |
| Unclassified *Lachnospiraceae* | | | 0.36 | 0.96 | | 0.388 | | 0.289 |
| *Collinsella* | | | 0.20 | 0.60 | | 0.355 | | 0.343 |
| *Desulfovibrio* | | | 0.36 | 0.32 | | 0.082 | | 0.653 |
| Unclassified *Clostridiaceae* | | | 0.62 | 0.04 | | 0.189 | | 0.048 |
| *Sutterella* | | | 0.18 | 0.37 | | 0.168 | | 0.425 |
| *Phascolarctobacterium* | | | 0.29 | 0.21 | | 0.102 | | 0.549 |
| *Campylobacter* | | | 0.22 | 0.17 | | 0.063 | | 0.540 |
| *Oscillospira* | | | 0.32 | 0.05 | | 0.069 | | 0.015 |
| Unclassified *GMD14H09* | | | 0.37 | 0.005 | | 0.169 | | 0.124 |
| Unclassified *Desulfovibrionaceae* | | | 0.28 | 0.05 | | 0.058 | | 0.005 |
| Unclassified *Alphaproteobacteria* | | | 0.30 | 0.004 | | 0.096 | | 0.050 |
| Unclassified *p-2534-18B6* | | | 0.29 | 0.001 | | 0.119 | | 0.110 |

^1^Data are presented as least square means ± SEM; *n* = 8 pigs per dietary treatment in the stomach, cecum, proximal colon, and mid colon; *n* = 7 pigs in the CON diet group and *n* = 4 pigs in the TGS diet group in the ileum.
